# Correction: RAB-10-Dependent Membrane Transport Is Required for Dendrite Arborization

**DOI:** 10.1371/journal.pgen.1006319

**Published:** 2016-09-09

**Authors:** The PLOS Genetics Staff

## Notice of Republication

This article was republished on October 21, 2015, to correct an XML error causing the 4^th^ author’s name, Yuh Nung Jan, to be indexed incorrectly. The publisher apologizes for the error. The correct citation is: Zou W, Yadav S, DeVault L, Jan YN, Sherwood DR (2015) RAB-10-Dependent Membrane Transport Is Required for Dendrite Arborization. PLoS Genet 11(9): e1005484. doi:10.1371/journal.pgen.1005484

## Supporting Information

S1 File Originally published, uncorrected article. (PDF)

S2 File Republished, corrected article. (PDF)

## References

[1] Zou W, Yadav S, DeVault L, Jan YN, Sherwood DR (2015) RAB-10-Dependent Membrane Transport Is Required for Dendrite Arborization. PLoS Genet 11(9): e1005484 doi: 10.1371/journal.pgen.1005484 26394140 10.1371/journal.pgen.1005484 PMC4578882

