# Supplementary figures and images for: RAB-10-Dependent Membrane Transport Is Required for Dendrite Arborization
Source: PLoS Genet. 2015 Sep 22;11(9):e1005484. doi: 10.1371/journal.pgen.1005484 (PMC4578882; doi:10.1371/journal.pgen.1005484)

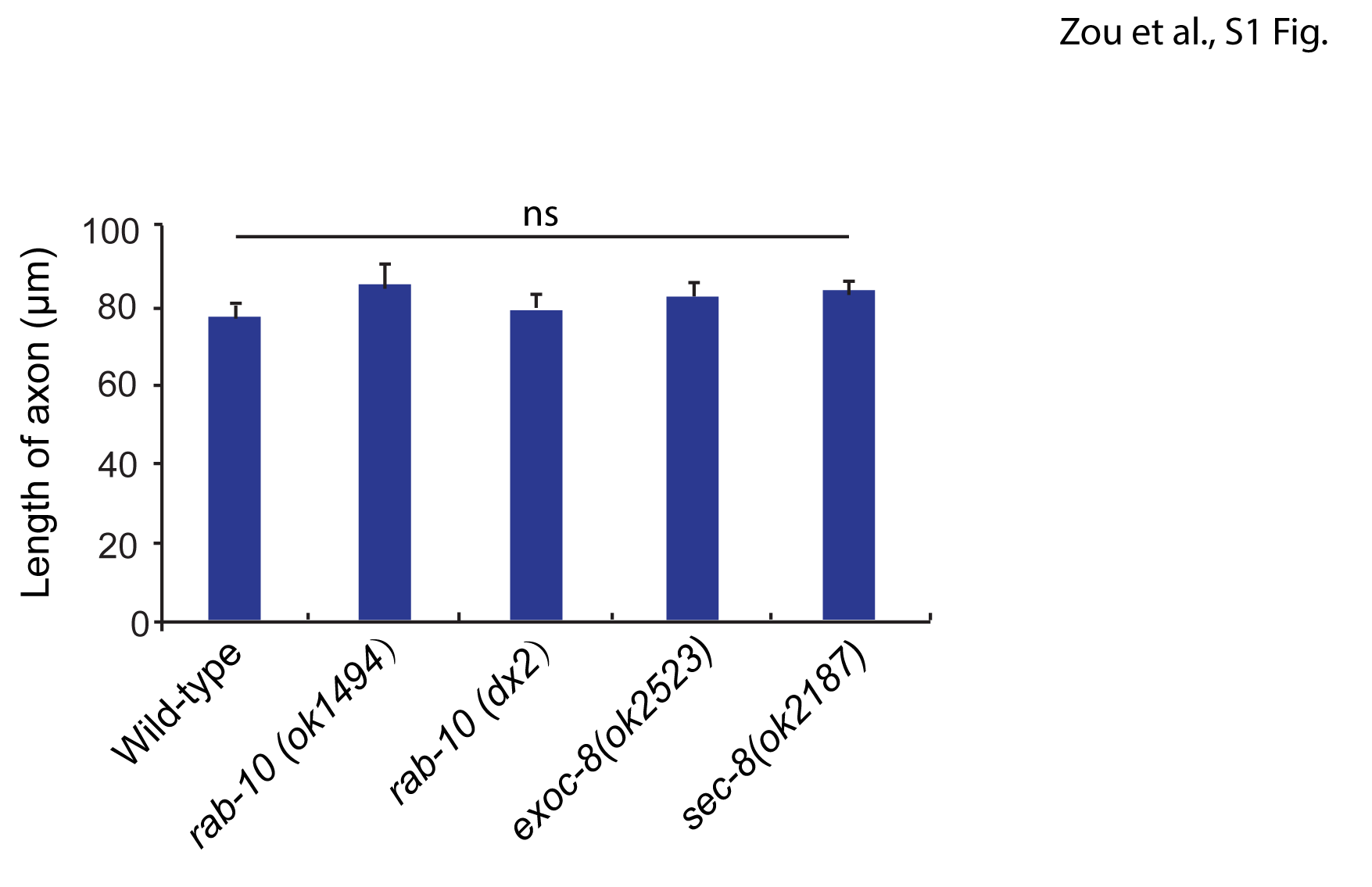

Supplement: S1 Fig — Length of axons was quantified for wild-type, rab-10(ok1494), rab-10(dx2), exoc-8(ok2523) and sec-8(ok2187) worms from maximum intensity projections of z-stacks using Image J. Axons were visualized using the wdIs51 strain. At least 15 animals were quantified for each genotype. A one-way ANOVA was used to compare wild-type and mutant animals. Error bars report ±SEM. ns: not significant. (TIF) [file pgen.1005484.s001.tif]

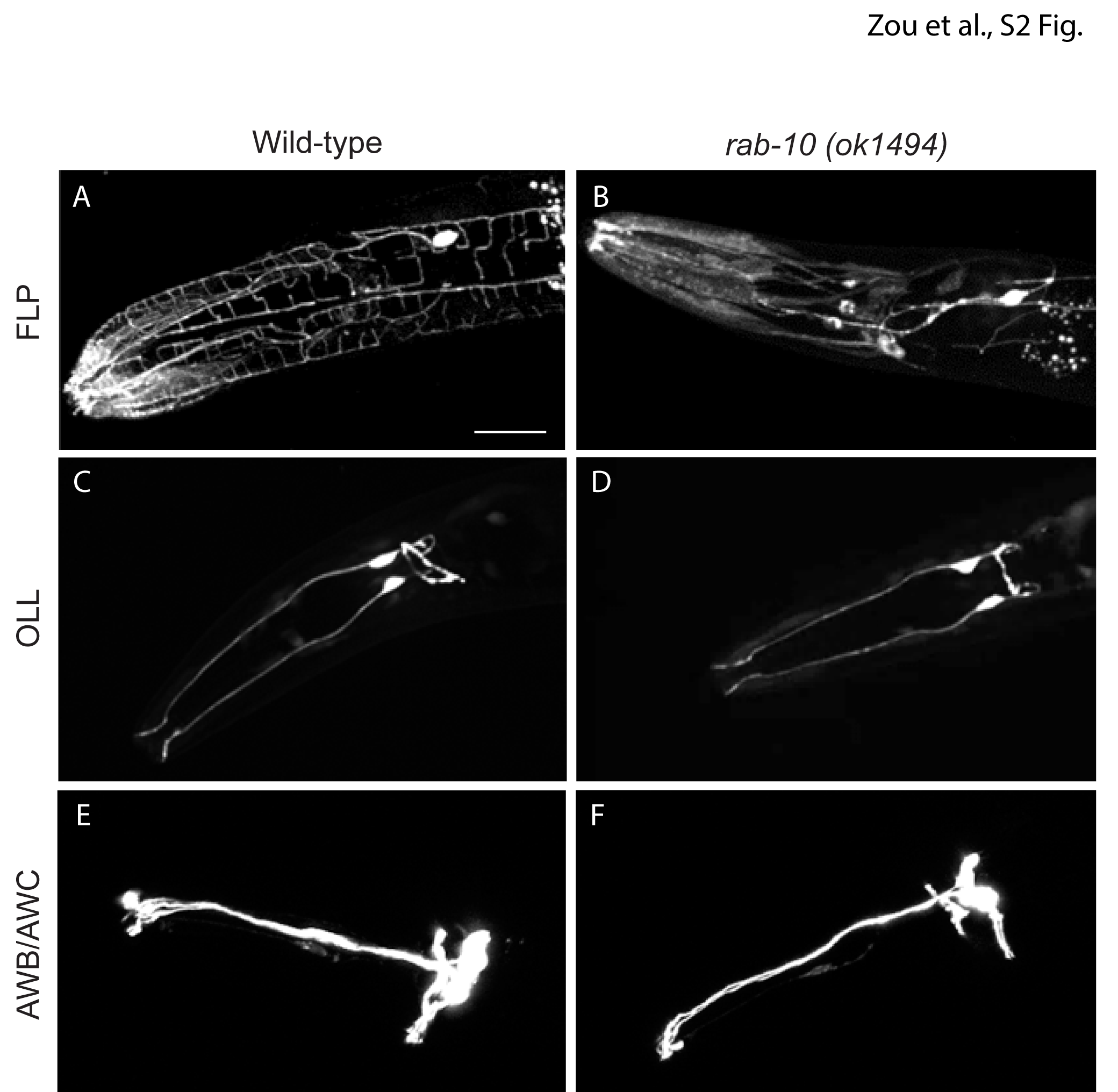

Supplement: S2 Fig — Morphology of (A, B) FLP neuron, (C, D) OLL neuron and (E, F) AWB and AWC neurons in wild-type (A, C, and E) and rab-10(ok1494) mutant animals (B, D and F) are shown. All images are maximum z projections. L4 or young adult stage animals were examined. Scale bar, 20 μm. (TIF) [file pgen.1005484.s002.tif]

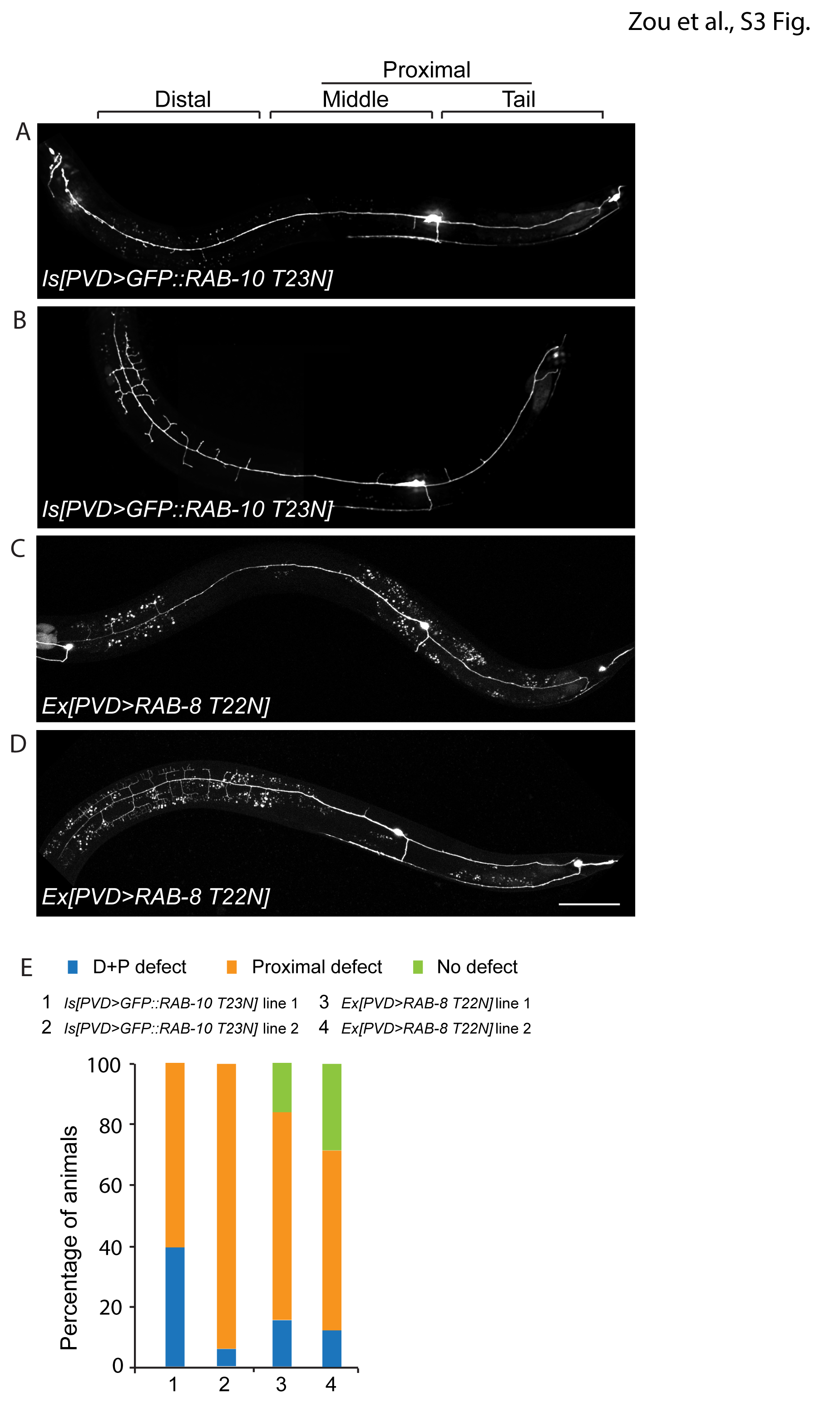

Supplement: S3 Fig — (A-D) Images showing the morphology of PVD neuron in (A, B) transgenic animals carrying Is[ser2prom3>gfp::rab-10 DN] and (C, D) transgenic animals carrying Ex[ser2prom3>rab-8 DN]. PVD morphology was visualized using the PVD>gfp marker strain wdIs51. All images are maximum z projections. L4 stage animals were examined. Scale bar, 50 μm. (E) Quantification of animals with wild-type or defective PVD dendrite morphology. Two independent lines were quantified for dominant negative RAB-10 and RAB-8, respectively. At least 30 worms were examined. D+P defect means that dendrite arborization in both distal and proximal regions were reduced (shown in A and C). Proximal defect means that only dendrites in the proximal region were affected (shown in B and D). No defect means the morphology of PVD dendrite arbors were indistinguishable from that of wild-type worms. (TIF) [file pgen.1005484.s003.tif]

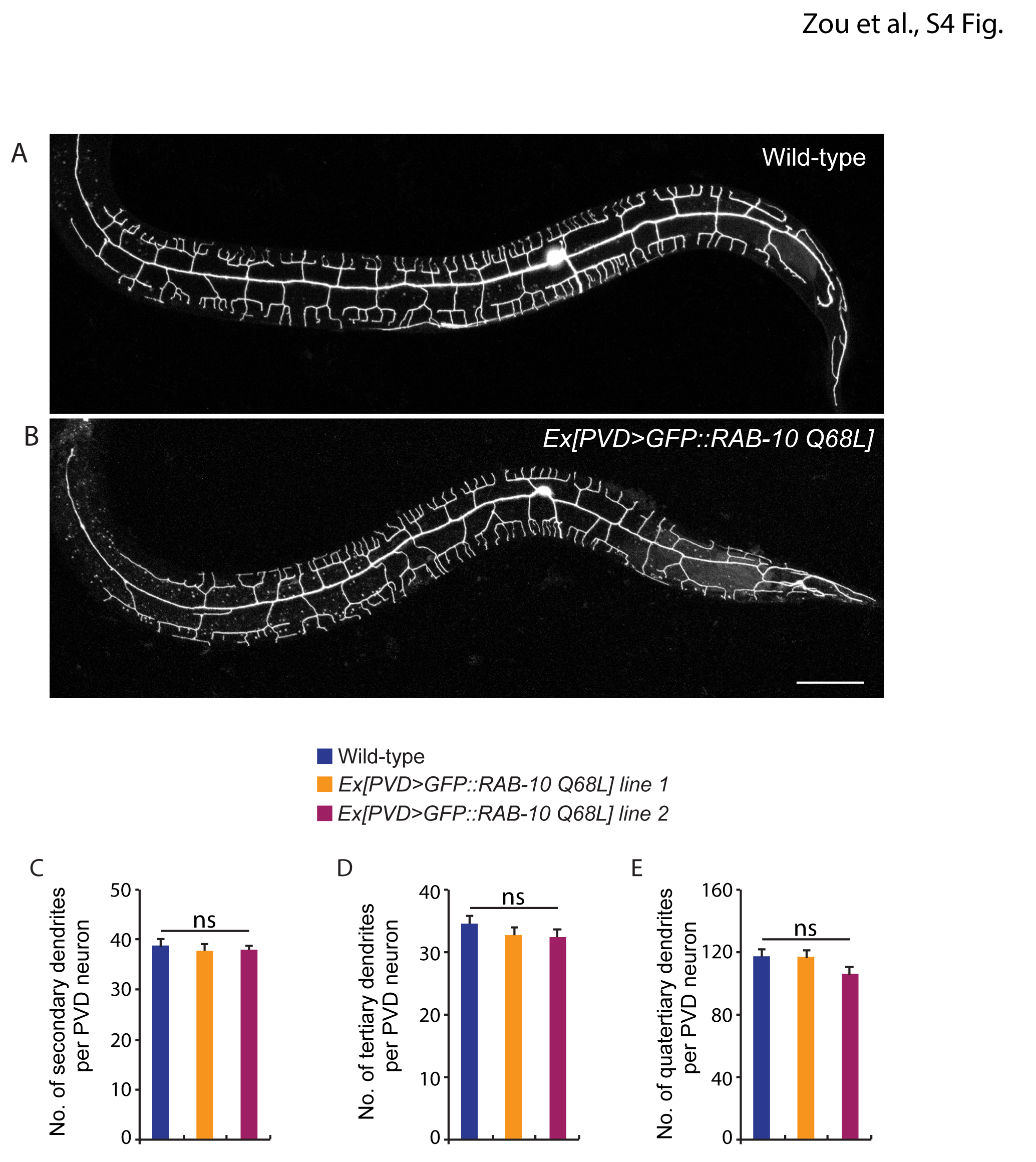

Supplement: S4 Fig — (A-B) Fluorescence images show the morphology of PVD neuron using the PVD>myr-gfp marker strain wyIs592 in (A) wild-type and (B) GFP::RAB-10 Q68L over-expression. Both images are maximum intensity projections of z-stacks. L4 stage animals were examined. Scale bar, 50 μm. (C-E) Quantification of number of (C) secondary dendrites, (D) tertiary dendrites and (E) quaternary dendrites per PVD neuron in the above three genotypes (n = 12 for each strain). A one-way ANOVA was used to compare wild-type and RAB-10 Q68L over-expressing lines. Error bars report ±SEM. ns: not significant. (TIF) [file pgen.1005484.s004.tif]

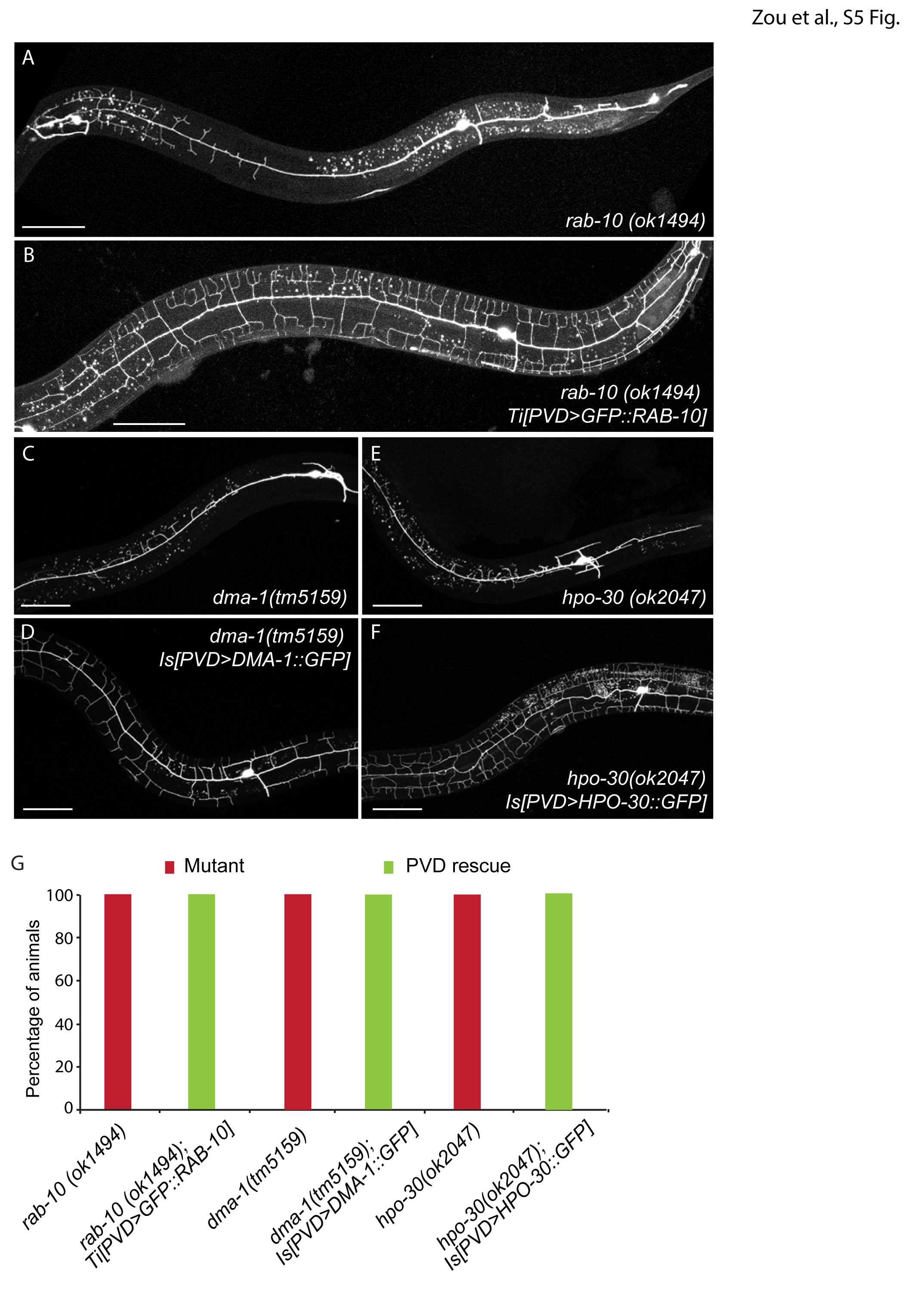

Supplement: S5 Fig — (A-F) Fluorescence images show the morphology of PVD neuron using the PVD>GFP marker strain wdIs51 in (A) rab-10(ok1494), (B) rab-10(ok1494); Ti[ser2prom3>gfp::rab-10], (C) dma-1(tm5159), (D) dma-1(tm5159); Is[ser2prom3>dma-1::gfp], (E) hpo-30(ok2047), and (F) hpo-30(ok2047); Is[ser2prom3>hpo-30::gfp]. All images are maximum intensity projections of z-stacks. L4 or young adult stage animals were examined. Scale bars, 50 μm. (G) Quantification of cell-autonomous rescue of dendritic morphogenesis defect by cell-specific expression of gfp::rab-10, dma-1::gfp and hpo-30::gfp transgenes. At least 30 animals were quantified for each genotype. (TIF) [file pgen.1005484.s005.tif]

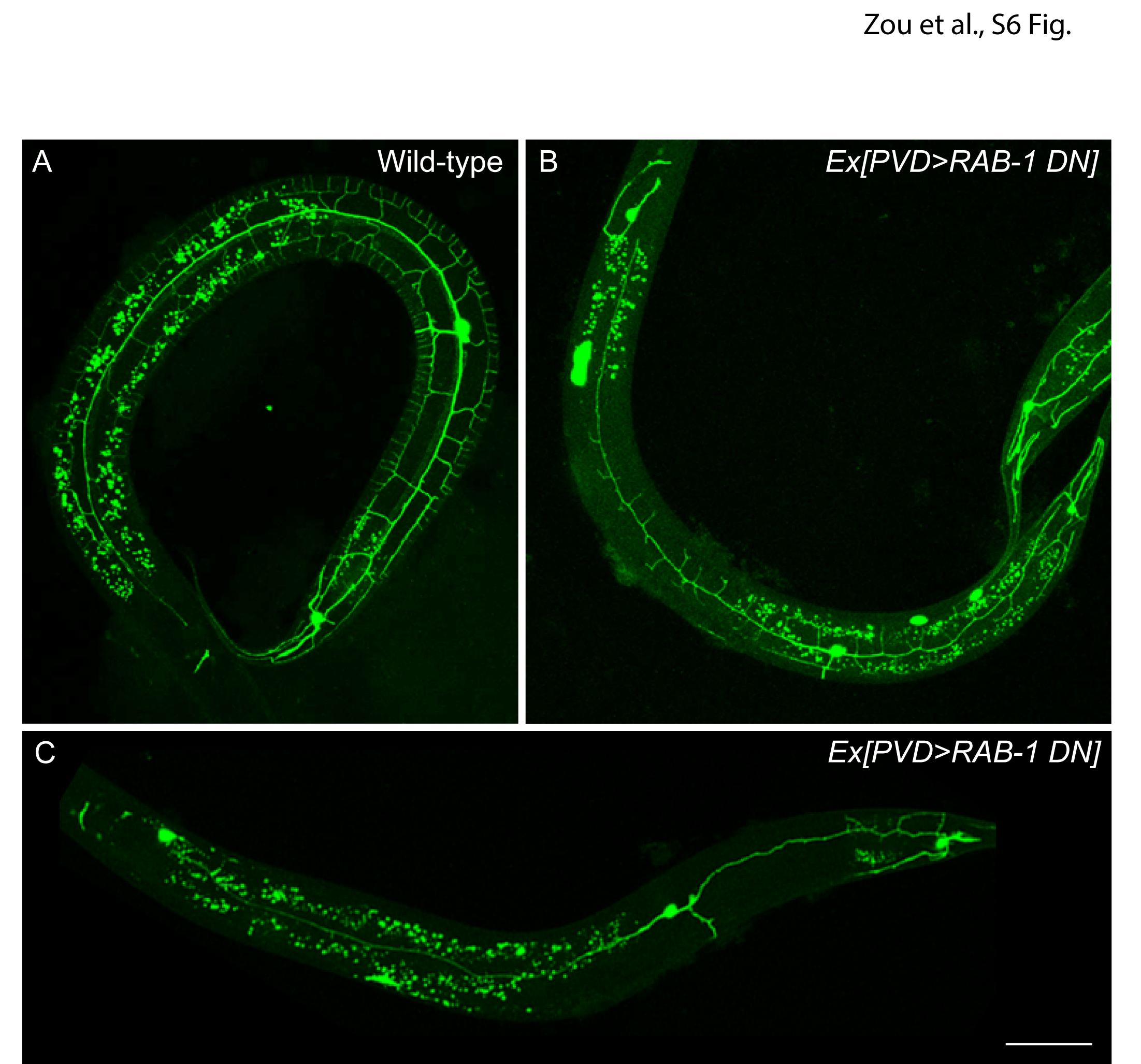

Supplement: S6 Fig — (A-C) PVD morphology was visualized using the strain wdIs51. Fluorescence images show the morphology of PVD neuron in (A) wild type, (B and C) ser2prom3>rab-1 DN transgenic animals. See Table 1 for quantification of defect. All images are maximum intensity projections of z-stacks. L4 or young adult stage animals were examined. Scale bar, 50 μm. (TIF) [file pgen.1005484.s006.tif]

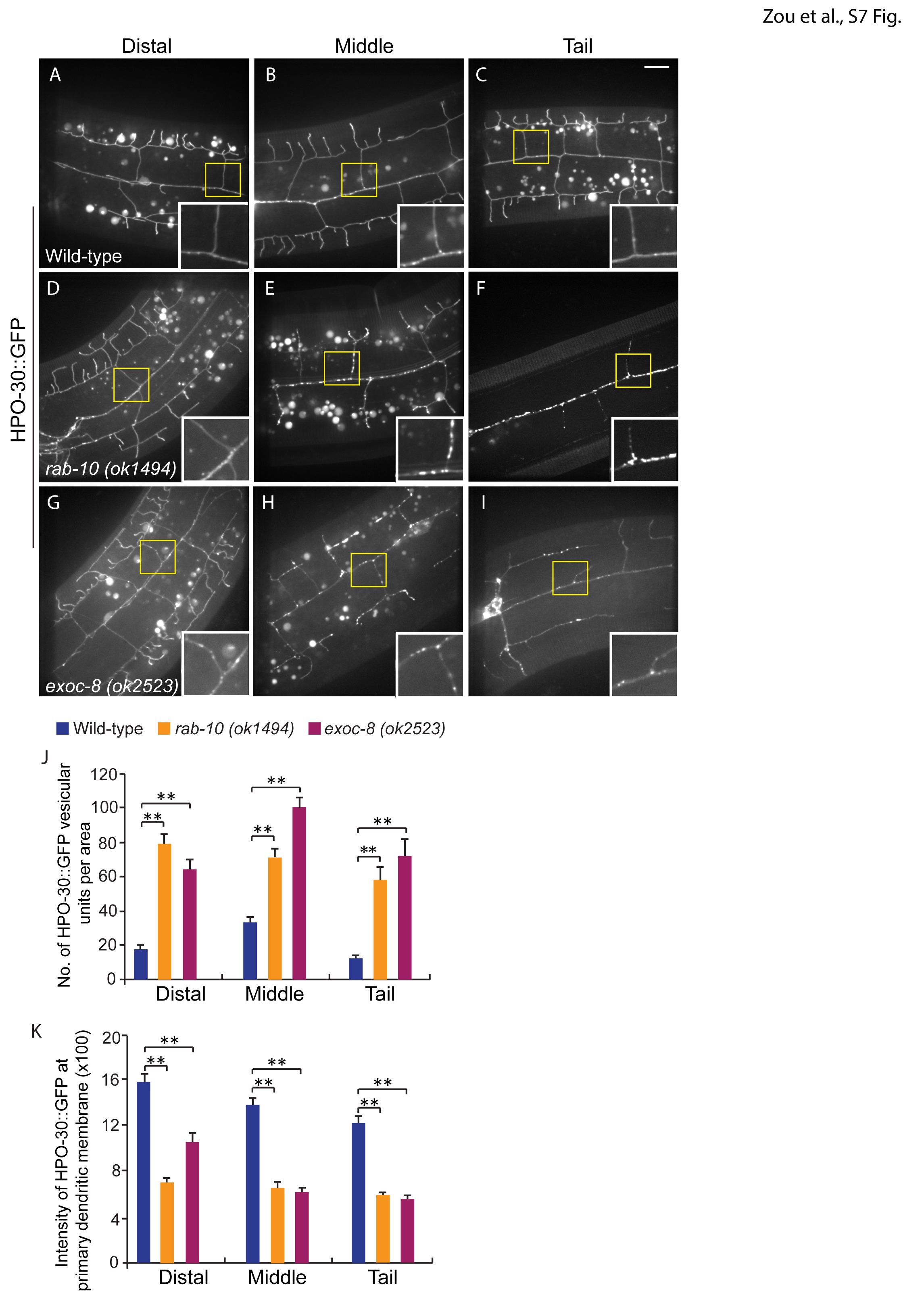

Supplement: S7 Fig — (A-I) Fluorescence images show subcellular localization of HPO-30::GFP in (A-C) wild-type, (D-F) rab-10 (ok1494) and (G-I) exoc-8 (ok2523) in the distal, middle and tail regions, respectively. All images are maximum intensity projections of z-stacks. L4 stage animals were examined. Insets are enlarged 2.5 fold. Scale bar, 10 μm. (J) Quantification of number of HPO-30::GFP vesicles/vesicle clusters per area (76.8μm x 76.8μm). 10 animals at the L4 stage were quantified for each genotype. (K) Quantification of the intensity of HPO-30::GFP at the surface of the primary dendrites in wild-type, rab-10 (ok1494) and exoc-8 (ok2523) mutant worms in the distal, middle, and tail regions, respectively. 10 animals at the L4 stage were quantified for each genotype. A one-way ANOVA followed by post-hoc comparisons using the Dunnett’s test was used to compare wild-type and mutant animals. **: P<0.01. Error bars report ±SEM. (TIF) [file pgen.1005484.s007.tif]

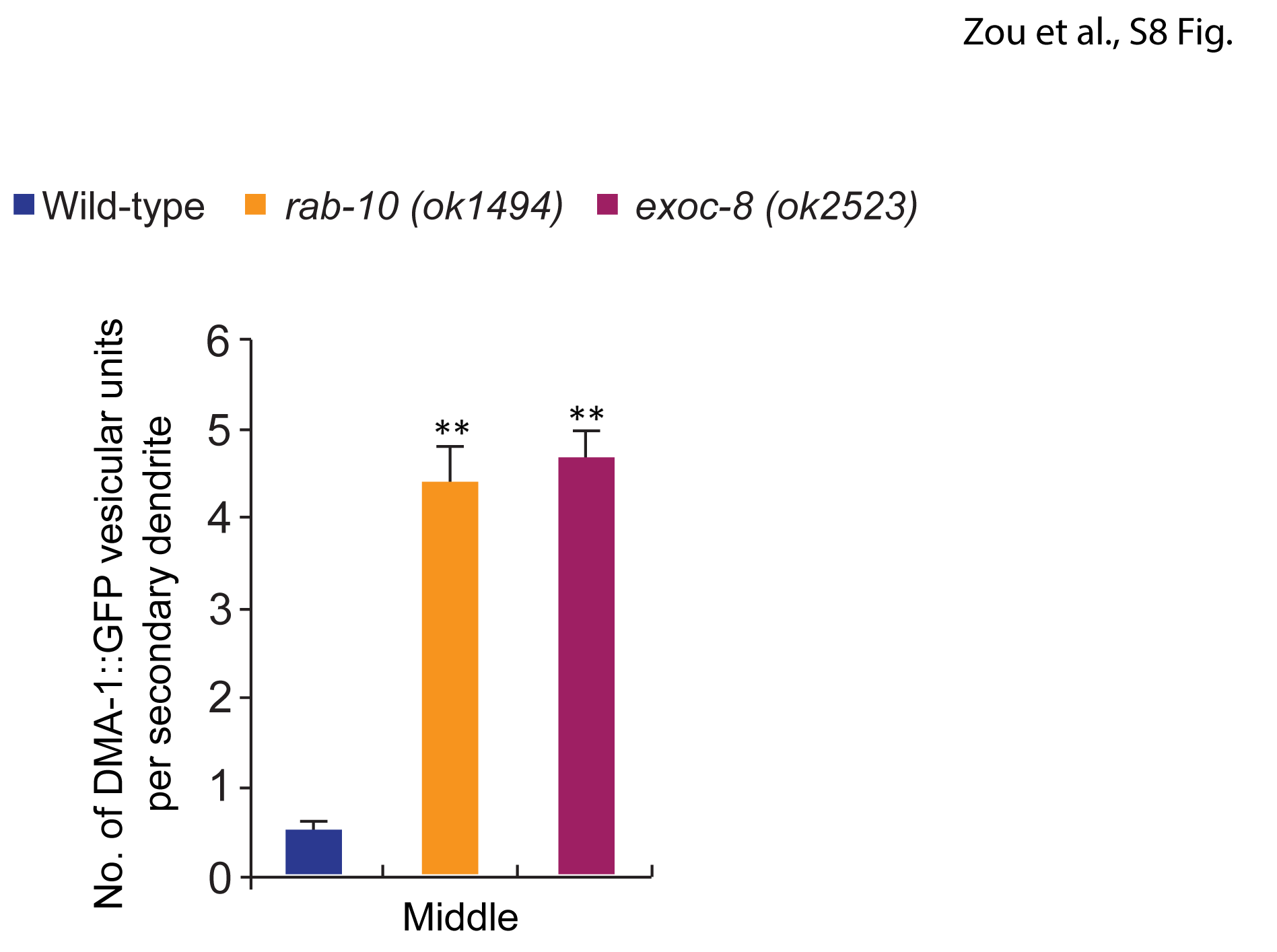

Supplement: S8 Fig — Number of vesicles or vesicle clusters labeled by DMA-1::GFP was quantified. At least 60 secondary dendrites were quantified for each genotype. A one-way ANOVA followed by post-hoc comparisons using the Dunnett’s test was used to compare wild-type and mutant animals. **: P<0.01. Error bars report ±SEM. (TIF) [file pgen.1005484.s008.tif]

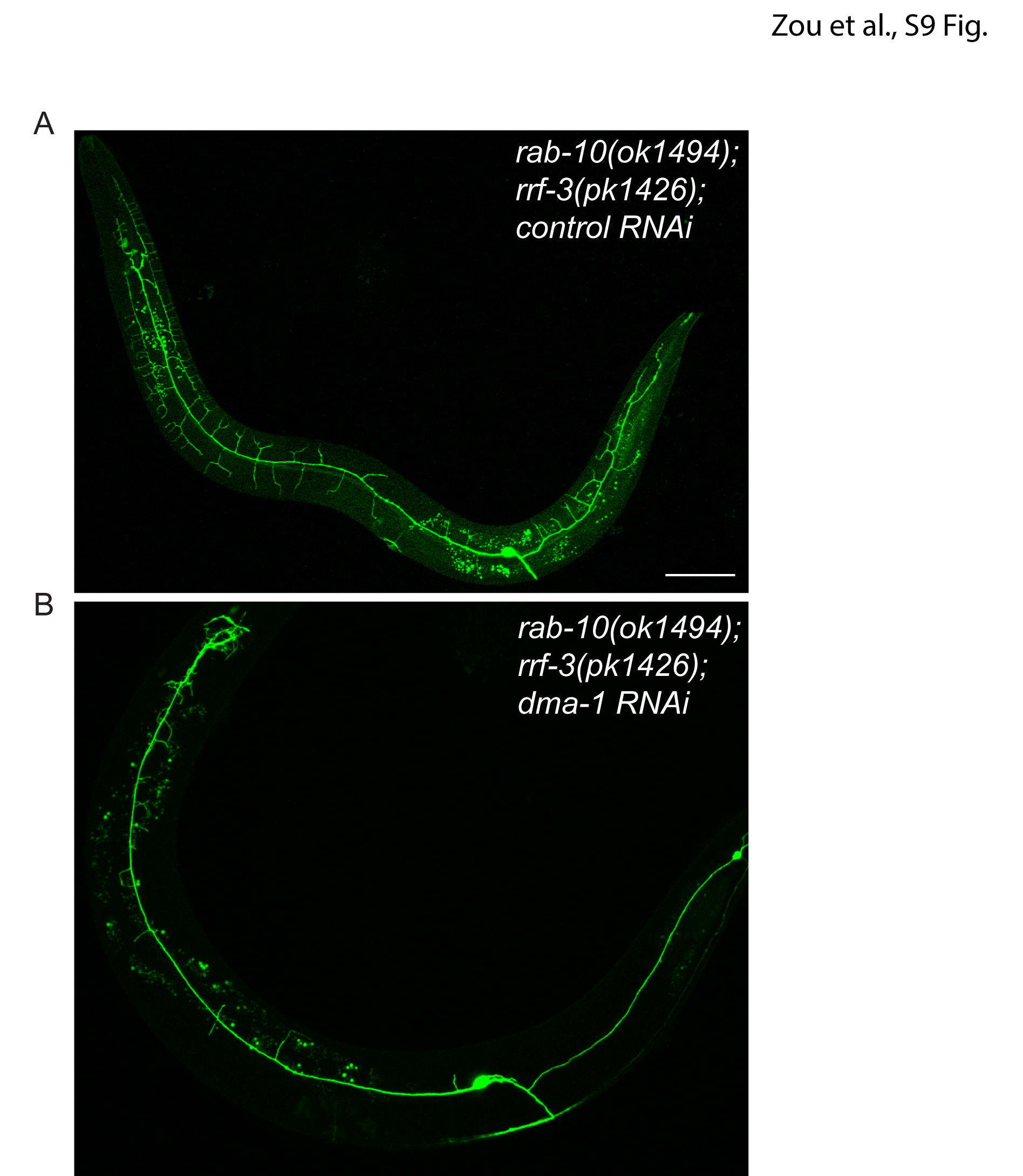

Supplement: S9 Fig — (A-B) Fluorescence images show the morphology of PVD neuron using the PVD>gfp marker strain wdIs51 in rab-10(ok1494); rrf-3(pk1426) fed with (A) control RNAi strain (harboring L4440 empty vector) and (B) dma-1 RNAi strain. Both images are maximum intensity projections of z-stacks. L4 or young adult stage animals were examined. Scale bar, 50 μm. (TIF) [file pgen.1005484.s009.tif]

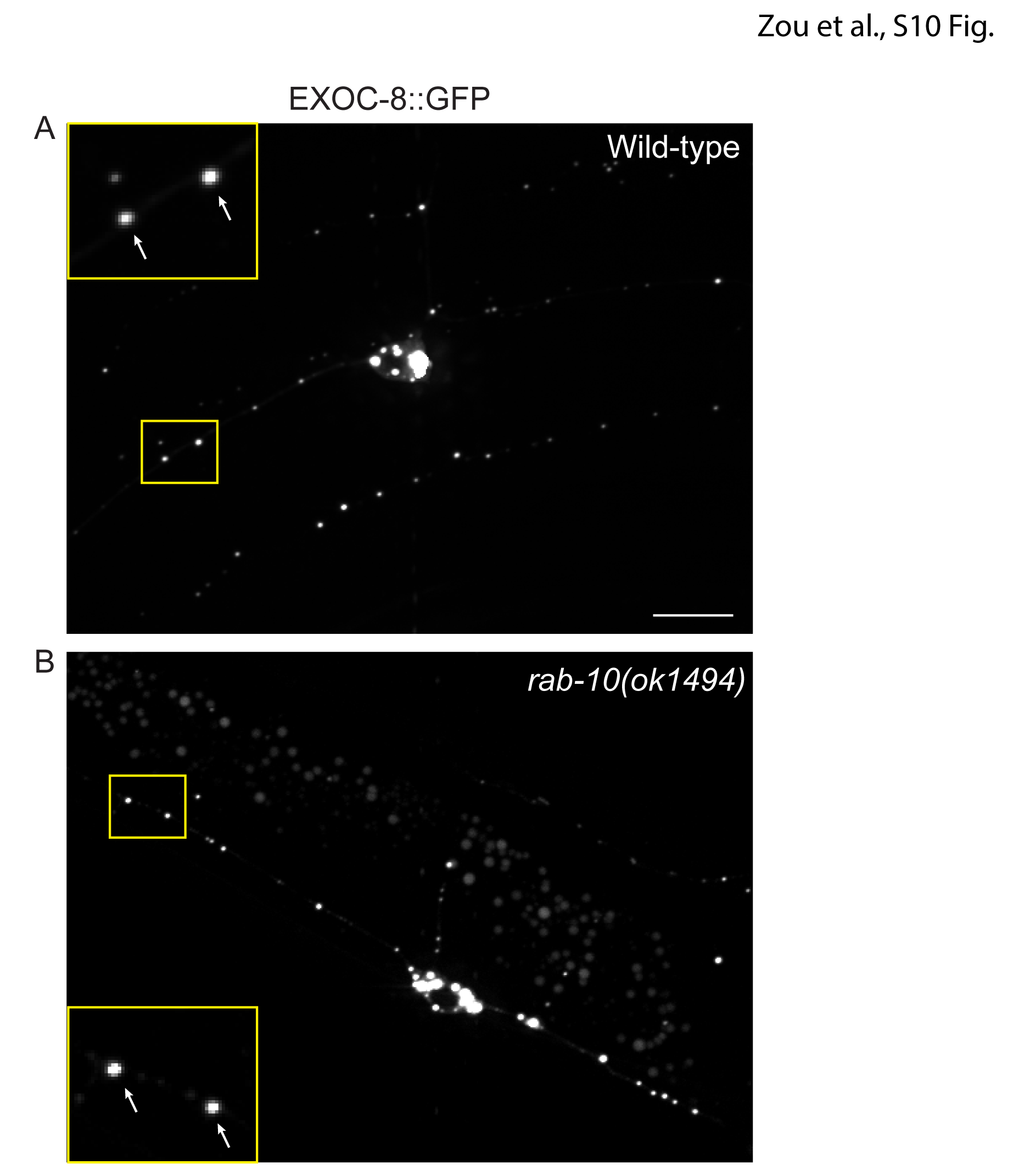

Supplement: S10 Fig — (A-B) Fluorescence images showing EXOC-8::GFP in (A) wild-type and (B) rab-10 (ok1494) mutant. Both images are maximum intensity projections of z-stacks. L4 or young adult stage animals were examined. Scale bars, 10 μm. The inset images are enlarged views (2.5 fold) of the regions indicated by the boxes. Arrows indicate vesicles labeled by EXOC-8::GFP reporter. (TIF) [file pgen.1005484.s010.tif]

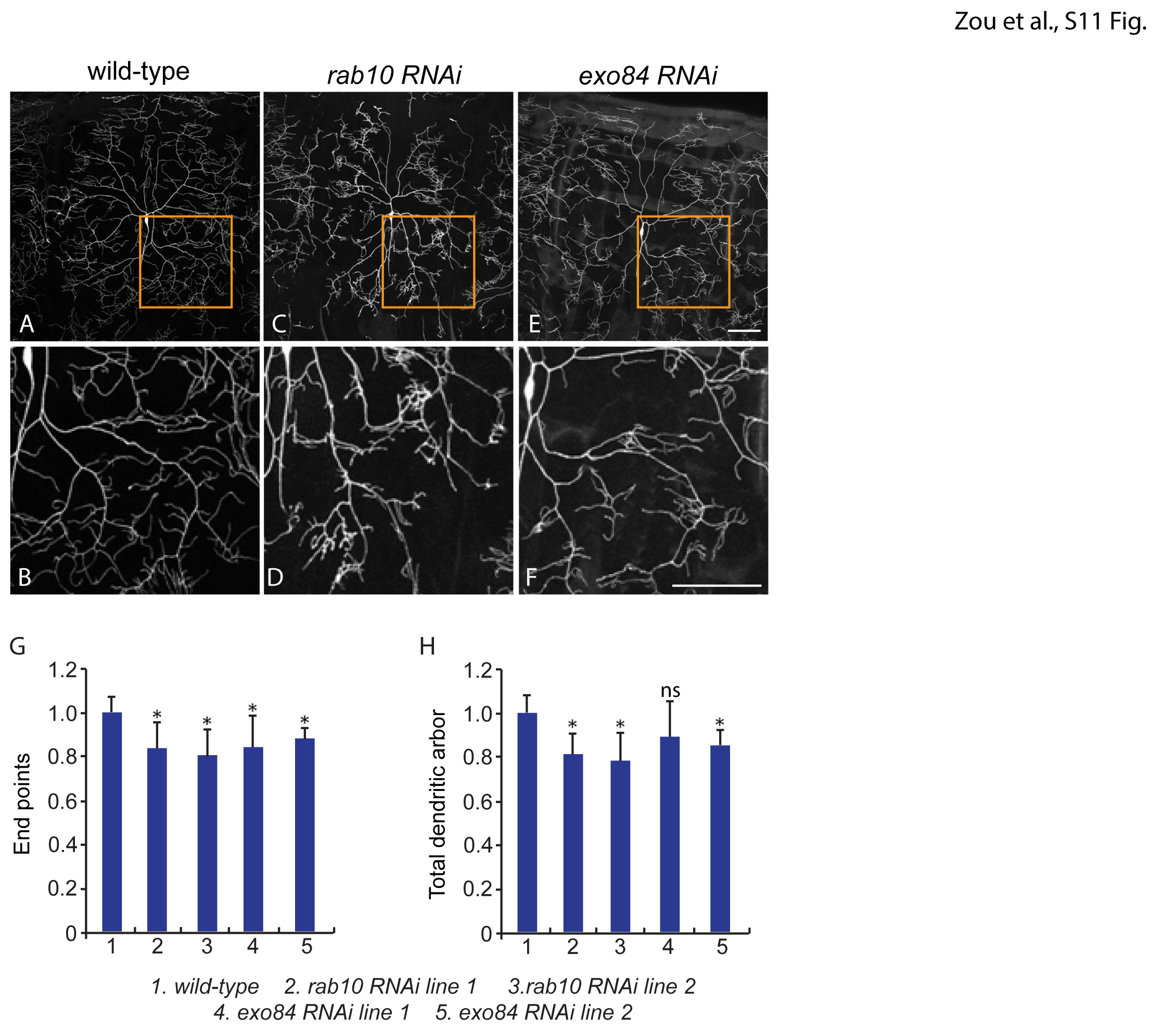

Supplement: S11 Fig — (A-F) Fluorescence images showing expression of a reporter ppk-tdgfp for the Drosophila class IV dendritic arborization neurons in (A and B) wild type, (C and D) rab10 RNAi and (E and F) exo84 RNAi treated animals. The images shown in (B, D and F) are magnified 2.5 fold from the boxed regions shown directly above. Scale bars, 100 μm. (G-H) Quantification of the number of (G) end points and (H) total dendritic arbor in wild type, rab10 RNAi (2 independent lines) and exo84 RNAi (2 independent lines) in Drosophila class IV dendritic arborization neurons. At least 7 neurons were quantified for each genotype. A one-way ANOVA followed by post-hoc comparisons using the Holm-Sidak test was used to compare wild-type and mutant animals. *: P<0.05; ns: not significant. Error bars report ±SD. (TIF) [file pgen.1005484.s011.tif]

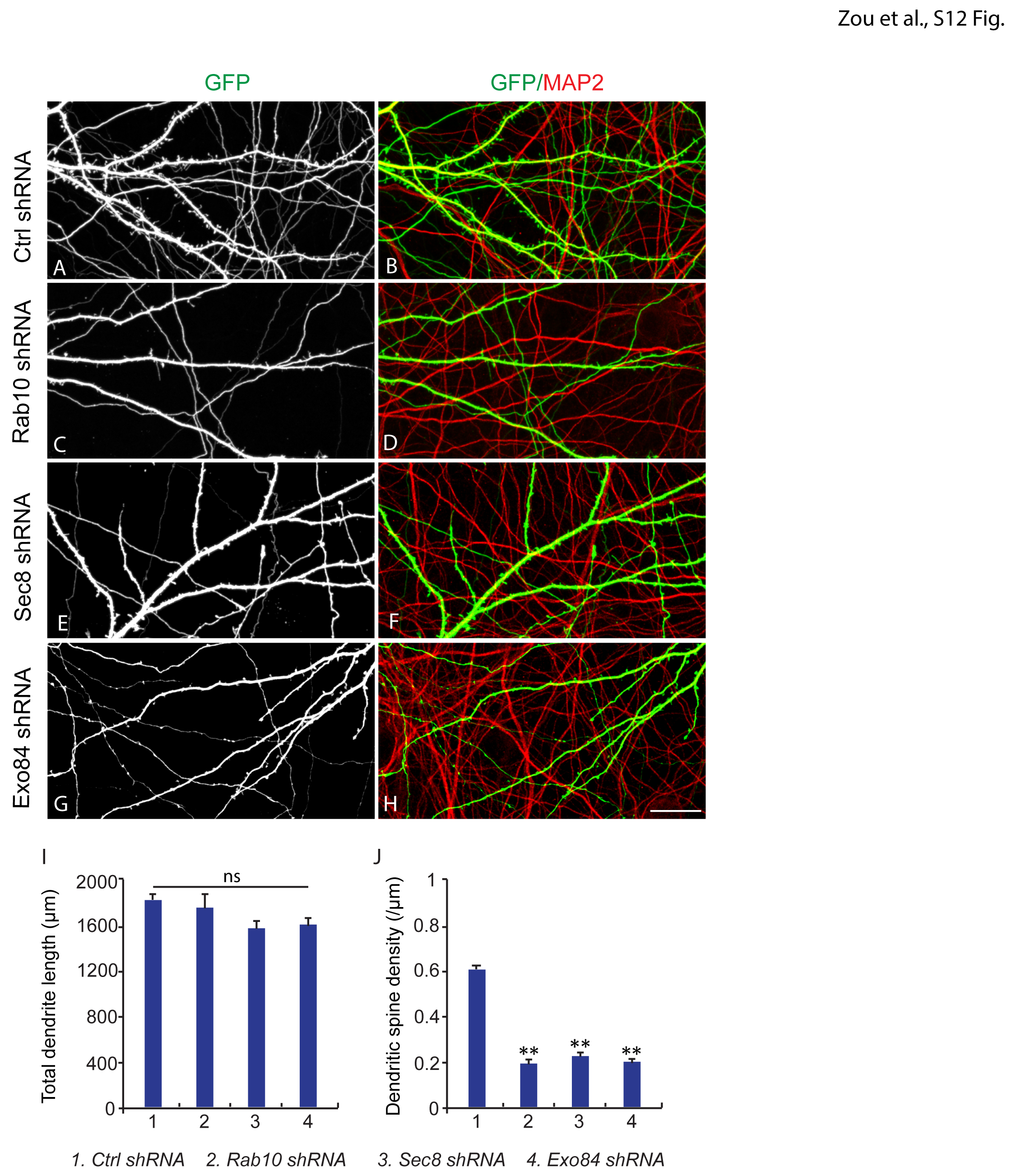

Supplement: S12 Fig — (A-H) Confocal fluorescence maximum projected images of cultured rat hippocampal neurons transfected with (A and B) control shRNA, (C and D) shRNA against Rab10 (E and F) shRNA against Sec8 and (G and H) shRNA against Exo84. Immunolocalization was conducted using antibodies against GFP (neuron, green) and MAP2 (dendrite, red). Scale bars, 20 μm. (I-J) Quantification of (I) total dendrite length at DIV9 and (J) dendritic spine density at DIV16 in control shRNA, Rab10 shRNA, Sec8 shRNAi and Exo84 shRNA transfected rat hippocampal neurons. 10 neurons were quantified for each genotype. A one-way ANOVA followed by post-hoc comparisons using the Dunnett’s test was used to compare wild-type and mutant animals. ns: not significant; **: P<0.01. Error bars report ±SEM. (TIF) [file pgen.1005484.s012.tif]
