# Supplementary material for: RAB-10-Dependent Membrane Transport Is Required for Dendrite Arborization
Source: PLoS Genet. 2015 Sep 22;11(9):e1005484. doi: 10.1371/journal.pgen.1005484 (PMC4578882; doi:10.1371/journal.pgen.1005484)
Supplement: S1 Table — (DOCX) [file pgen.1005484.s014.docx]

**S1 Table. Transgenes used in this study**

| **Allele** | **Constructs** | **Note** | **Reference** |
| --- | --- | --- | --- |
| *wdIs51* | *F49H12.4*>*gfp*  *and unc-119(+)* | To visualize the morphology of PVD neuron | [[1](#_ENREF_1)] |
| *otIs138* | *ser2prom3*>*gfp*  *and rol-6(d)* | To visualize the morphology of PVD and OLL neurons | [[2](#_ENREF_2)] |
| *wyIs592* | *ser2prom3*>*myr-gfp*, and *odr-1*>*rfp* | To visualize the morphology of PVD, OLL, AWB and AWC neurons | Kindly provided by Dr. Kang Shen |
| *hmIs4* | *Pdes-2*>*des-2::gfp* | To visualize the morphology of FLP and PVD neurons | [[3](#_ENREF_3)] |
| *qyIs307* | *ser2prom3>gfp::rab-10, ser2prom3>mcherry,* and *unc-119 (+)* | To examine the subcellular localization of RAB-10 in PVD neuron | This study |
| *qyIs368* | *ser2prom3*>*dma-1::gfp* and *unc-119(+)* | To examine the subcellular localization of DMA-1 in PVD neuron | This study |
| *qyIs366* | *ser2prom3*>*hpo-30::gfp* and *unc-119(+)* | To examine the subcellular localization of HPO-30 in PVD neuron | This study |
| *qyIs375* | *ser2prom3>exoc-8::gfp, Pmyo-2>mcherry* and *unc-119(+)* | To examine the subcellular localization of EXOC-8 in PVD neuron | This study |
| *qyTi1* | *ser2prom3>zf1::gfpnovo2::*  *rab-10* and *unc-119 (+)* | To examine the subcellular localization of RAB-10 in PVD neuron | This study |
| *qyEx525* | *ser2prom3*>*mcherry::fapp1-ph* and *Podr-1>gfp* | To examine Golgi in PVD neuron | This study |
| *qyEx526* | *ser2prom3*>*mcherry::rab-5* and *Podr-1>gfp* | To examine early endosomes in PVD neuron | This study |
| *qyEx379* | *ser2prom3>mcherry::rab-10, ser2prom3>exoc-8::gfp* and *unc-119(+)* | To examine RAB-10 and EXOC-8 co-localization in PVD neuron | This study |
| *qyEx484* | *ser2prom3>mcherry::rab-10, ser2prom3>dma-1::gfp* and *unc-119(+)* | To examine RAB-10 and DMA-1 co-localization in PVD neuron | This study |
| *qyEx485* | *ser2prom3>mcherry::rab-10, ser2prom3>hpo-30::gfp* and *unc-119(+)* | To examine RAB-10 and HPO-30 co-localization in PVD neuron | This study |
| *qyEx361* | *ser2prom3*>*gfp::rab-10* and *Pmyo-2>mcherry* | To over-express wild-type RAB-10 in the PVD neuron of *rab-10* mutant | This study |
| *qyEx398* | *ser2prom3*>*gfp::rab-10* and *Pmyo-2>mcherry* | To over-express wild-type RAB-10 in the PVD neuron of *rab-10* mutant | This study |
| *qyEx486* | *ser2prom3*>*gfp::rab-10 (T23N)* and *Pmyo-2>mcherry* | To over-express dominant-negative RAB-10 in the PVD neuron of *rab-10* mutant | This study |
| *qyEx487* | *ser2prom3*>*gfp::rab-10 (T23N)* and *Pmyo-2>mcherry* | To over-express dominant-negative RAB-10 in the PVD neuron of *rab-10* mutant | This study |
| *qyEx488* | *ser2prom3*>*gfp::rab-10* (Q68L) and *Pmyo-2>mcherry* | To over-express constitutively- active RAB-10 in the PVD neuron of *rab-10* mutant | This study |
| *qyEx489* | *ser2prom3*>*gfp::rab-10* (Q68L) and *Pmyo-2>mcherry* | To over-express constitutively- active RAB-10 in the PVD neuron of *rab-10* mutant | This study |
| *qyEx358* | *ser2prom3*>*gfp::rab-10* (Q68L), *ser2prom3>myr-mcherry* and *unc-119(+)* | To over-express constitutively- active RAB-10 in the PVD neuron of wild-type animals | This study |
| *qyEx360* | *ser2prom3*>*gfp::rab-10* (Q68L), *ser2prom3>myr-mcherry* and *unc-119(+)* | To over-express constitutively- active RAB-10 in the PVD neuron of wild-type animals | This study |
| *qyIs318* | *ser2prom3*>*gfp::rab-10* (T23N); *ser2prom3*>*myr-mcherry* and *unc-119(+)* | To over-express dominant –negative RAB-10 in the PVD neuron of wild-type animals | This study |
| *qyIs319* | *ser2prom3*>*gfp::rab-10* (T23N); *ser2prom3*>*myr-mcherry* and *unc-119(+)* | To over-express dominant –negative RAB-10 in the PVD neuron of wild-type animals | This study |
| *qyEx490* | *ser2prom3*> *rab-8* (T22N) and *Pmyo-2>mcherry* | To over-express dominant –negative RAB-8 in the PVD neuron of wild-type animals | This study |
| *qyEx491* | *ser2prom3*> *rab-8* (T22N) and *Pmyo-2>mcherry* | To over-express dominant –negative RAB-8 in the PVD neuron of wild-type animals | This study |
| *qyEx492* | *ser2prom3*> *rab-1* DN and *Punc-122>rfp* | To over-express dominant –negative RAB-1 in the PVD neuron of wild-type animals | This study |
| *qyEx493* | *ser2prom3*>*rab-1* DN and *Punc-122>rfp* | To over-express dominant –negative RAB-1 in the PVD neuron of wild-type animals | This study |
| *qyEx494* | *ser2prom3*> *rab-5* DN and *Punc-122>rfp* | To over-express dominant –negative RAB-5 in the PVD neuron of wild-type animals | This study |
| *qyEx495* | *ser2prom3*> *rab-5* DN and *Punc-122>rfp* | To over-express dominant –negative RAB-5 in the PVD neuron of wild-type animals | This study |
| *qyEx496* | *ser2prom3*> *rab-11.1* DN and *Punc-122>rfp* | To over-express dominant –negative RAB-11.1 in the PVD neuron of wild-type animals | This study |
| *qyEx497* | *ser2prom3*> *rab-11.1* DN and *Punc-122>rfp* | To over-express dominant –negative RAB-11.1 in the PVD neuron of wild-type animals | This study |
| *qyEx369* | *ser2prom3>exoc-8::gfp, Pmyo-2>mcherry* and *unc-119(+)* | To over-express EXOC-8 in the PVD neuron | This study |
| *qyEx370* | *ser2prom3>exoc-8::gfp, Pmyo-2>mcherry* and *unc-119(+)* | To over-express EXOC-8 in the PVD neuron | This study |
| *qyEx498* | *ser2prom3>zif-1, ser2prom3>gfp* and *Pmyo-2>mcherry* | To over-express ZIF-1 in the PVD neuron | This study |
| *qyEx499* | *ser2prom3>zif-1, ser2prom3>gfp* and *Pmyo-2>mcherry* | To over-express ZIF-1 in the PVD neuron | This study |
| *qyEx527* | *Pnhr-81>Cas9, PU6>rab-10 sgRNA#1, PU6>rab-10 sgRNA#2, Pmyo-2>mcherry* and *Pmyo-3>mcherry* | To generate *rab-10* conditional knock-out in PVD neuron and other descendants derived from the seam cell lineage | This study |
| *qyEx528* | *Pnhr-81>Cas9, PU6>rab-10 sgRNA#1, PU6>rab-10 sgRNA#2, Pmyo-2>mcherry* and *Pmyo-3>mcherry* | To generate *rab-10* conditional knock-out in PVD neuron and other descendants derived from the seam cell lineage | This study |
| *qyEx529* | *Pnhr-81>Cas9, PU6>rab-10 sgRNA#1, PU6>rab-10 sgRNA#2, Pmyo-2>mcherry* and *Pmyo-3>mcherry* | To generate *rab-10* conditional knock-out in PVD neuron and other descendants derived from the seam cell lineage | This study |

1. Smith CJ, Watson JD, Spencer WC, O'Brien T, Cha B, et al. (2010) Time-lapse imaging and cell-specific expression profiling reveal dynamic branching and molecular determinants of a multi-dendritic nociceptor in C. elegans. Dev Biol 345: 18-33.

2. Aguirre-Chen C, Bulow HE, Kaprielian Z (2011) C. elegans bicd-1, homolog of the Drosophila dynein accessory factor Bicaudal D, regulates the branching of PVD sensory neuron dendrites. Development 138: 507-518.

3. Oren-Suissa M, Hall DH, Treinin M, Shemer G, Podbilewicz B (2010) The fusogen EFF-1 controls sculpting of mechanosensory dendrites. Science 328: 1285-1288.
