# Supplementary material for: RAB-10-Dependent Membrane Transport Is Required for Dendrite Arborization
Source: PLoS Genet. 2015 Sep 22;11(9):e1005484. doi: 10.1371/journal.pgen.1005484 (PMC4578882; doi:10.1371/journal.pgen.1005484)
Supplement: S2 Table — (DOCX) [file pgen.1005484.s015.docx]

**S2 Table. Plasmids and fusion PCR products used in this study**

| Name | Description | Used in which transgene |
| --- | --- | --- |
| pWZ8 | pPD49.26-*ser2prom3>gfp::rab-10* | *qyEx361* and *qyEx398* |
| pWZ9 | pPD49.26-*ser2prom3>gfp::rab-10 (T23N)* | *qyEx486, qyEx487, qyIs318* and *qyIs319* |
| pWZ10 | pPD49.26-*ser2prom3>gfp::rab-10 (Q68L)* | *qyEx488,*  *qyEx489, qyEx358* and *qyEx360* |
| pWZ11 | pPD49.26-*ser2prom3>gfp* | *qyEx498* and *qyEx499* |
| pWZ20 | pPD49.26-*ser2prom3>exoc-8::gfp* | *qyEx369*, *qyEx370, qyIs375* and *qyEx379* |
| pWZ25 | pPD49.26-*ser2prom3>mcherry::rab-10* | *qyEx379, qyEx484* and *qyEx485* |
| pWZ54 | pPD95.75-*ser2prom3>hpo-30::gfp* | *qyIs366* and *qyEx485* |
| pWZ55 | pPD95.75-*ser2prom3>dma-1::gfp* | *qyIs368* and *qyEx484* |
| pWZ70 | pPD49.26-*ser2prom3>rab-1(S25N)* | *qyEx492* and *qyEx493* |
| pWZ71 | pPD49.26-*ser2prom3>rab-5 (S33N)* | *qyEx494* and *qyEx495* |
| pWZ72 | pPD49.26-*ser2prom3>rab-8 (T22N)* | *qyEx490* and *qyEx491* |
| pWZ73 | pPD49.26-*ser2prom3>rab-11.1 (S25N)* | *qyEx496* and *qyEx497* |
| pWZ131 | pPD49.26-*ser2prom3>zif-1* | *qyEx498* and *qyEx499* |
| pWZ170 | *PU6*>*rab-10* sgRNA #1 | *qyEx527, qyEx528* and *qyEx529* |
| pWZ171 | *PU6*>*rab-10* sgRNA #2 | *qyEx527, qyEx528* and *qyEx529* |
| pWZ242 | pCFJ909-*ser2prom3*>*zf1::gfpnovo2::rab-10::unc-54* *3’utr* | *qyTi1* |
| pWZ243 | P*nhr-81*>*Cas9* | *qyEx527, qyEx528* and *qyEx529* |
| Fusion PCR#15 | *ser2prom3>mcherry::rab-5::let-858 3’utr* | *qyEx526* |
| Fusion PCR#58 | *ser2prom3>myr-mcherry::unc-54 3’utr* | *qyIs307, qyIs318* and *qyIs319* |
| Fusion PCR#61 | *ser2prom3>gfp::rab-10::unc-54 3’utr* | *qyIs307* |
| Fusion PCR#64 | *ser2prom3>mcherry::fapp1-ph::let-858 3’utr* | *qyEx525* |
