# Supplementary material for: RAB-10-Dependent Membrane Transport Is Required for Dendrite Arborization
Source: PLoS Genet. 2015 Sep 22;11(9):e1005484. doi: 10.1371/journal.pgen.1005484 (PMC4578882; doi:10.1371/journal.pgen.1005484)
Supplement: S3 Table — (DOCX) [file pgen.1005484.s016.docx]

**S3 Table. Primers used in this study**

| **Name** | **Sequence** | **Note** | **Used in which plasmid or fusion PCR product** |
| --- | --- | --- | --- |
| oWZ16 | tggttattccgaaacgctgtc | *ser2prom3* (1.6 kb) S-outer | PCR product #15, 58, 61 and 64 |
| oWZ15 | cattatgtgttgtgatgtcacaaaaatatgcc | *ser2prom3* (1.6 kb) AS | PCR product #15, 58, 61 and 64 |
| oWZ14 | gtcgacttcaactgtaggcg | *ser2prom3* (1.6 kb) S-inner | PCR product #15, 58, 61 and 64 |
| oWZ9 | ttgtgacatcacaacacataatgatggtctcaaagggtgaag | linker (23bp overlap with *ser-2prom3*)+*mcherry* (worm codon optimized) NS | PCR product #15 |
| oWZ50 | ttgtgacatcacaacacataatgatggtgagcaagggcgagg | linker (23bp overlap with *ser-2prom3*)+*mcherry* NS | PCR product #64 |
| oWZ5 | aaaagcggttagctccttcggtcc | *let-858 3’UTR* AS outer | PCR product #15 and 64 |
| oWZ6 | tctgtgactggtgagtactcaacc | *let-858* *3’UTR* AS inner | PCR product #15 and 64 |
| oWZ8 | ttgtgacatcacaacacataatgatgagtaaaggagaagaac | linker (23bp overlap with *ser-2prom3*)+*gfp* NS | PCR product # 61 |
| oWZ49 | ttgtgacatcacaacacataatgatgggttcctgtattggaaaa | linker (23bp overlap with *ser-2prom3*) + *myri* signal NS | PCR product #58 |
| oWZ45 | gaaacgcgcgagacgaaagggcccgt | *unc-54* 3'UTR AS outer | PCR product #58 and 61 |
| oWZ46 | aagggcccgtacggccgactagtagg | *unc-54* 3'UTR AS inner | PCR product #58 and 61 |
| oWZ29 | gc **aagctt** gtcgacttcaactgtaggcg | *ser2prom3* HindIII S | All plasmids with *ser2prom3* promoter |
| oWZ30 | gc **cccggg** tatgtgttgtgatgtcacaaaaatatgcc | *ser2prom3* XmaI AS | All plasmids with *ser2prom3* promoter |
| oWZ41 | gc **ggtacc** atggctcgccgaccgtatgac | *rab-10* NS KpnI | pWZ8, 9, 10 and 242 |
| oWZ102 | gc **gagctc** ctagcagcatcctccactgc | *rab-10* CAS SacI | pWZ8, 9, 10 and 242 |
| oWZ107 | cg **cccggg** atgccattaattatgtacaa | *hpo-30* NS SmaI | pWZ54 |
| oWZ163 | gc **accggt** accatactgctgtcatcgtcaatc | *hpo-30* CAS AgeI without stop codon, 2bp added to make *hpo-30* in-frame with downstream *gfp* | pWZ54 |
| oWZ130 | cg **cccggg** atgatttcatacattttgc | *dma-1* NS SmaI | pWZ55 |
| oWZ164 | gc **accggt** *ac*aatgccaaaataggatgatc | *dma-1* CAS AgeI without stop codon, 2bp added to make *dma-1* in-frame with downstream *gfp* | pWZ55 |
| oWZ117 | cg **cccggg** atgctttcaaatgaggatgg | *exoc-8* NS XmaI | pWZ20 |
| oWZ118 | gc **ggtacc** aacatgagcaacttcttcatc | *exoc-8* CAS KpnI w/o stop codon | pWZ20 |
| oWZ156 | cg **cccggg** atggcagcaatgaaccctg | *rab-1* NS XmaI | pWZ70 |
| oWZ157 | gc **ggtacc** ttaacaacatccaccgctc | *rab-1* CAS KpnI with stop codon | pWZ70 |
| oWZ160 | cg **cccggg** atggccgcccgaaacgcagg | *rab-5 NS XmaI* | pWZ71 |
| oWZ53 | gc **ggtacc** ttatttacagcatgaaccct | *rab-5* CAS KpnI with stop codon | pWZ71 |
| oWZ158 | cg **cccggg** atggcaaaaacttacgactac | *rab-8* NS XmaI | pWZ72 |
| oWZ159 | gc **ggtacc** ttaaagcaaattgcagctcc | *rab-8* CAS KpnI with stop codon | pWZ72 |
| oWZ214 | cg **cccggg** atgggctctcgtgacgatga | *rab-11.1* NS XmaI | pWZ73 |
| oWZ215 | gc **ggtacc** ttatgggatgcaacactgcttc | *rab-11.1* CAS KpnI with stop codon | pWZ73 |
| oWZ258 | cg **cccggg** atgagtgagtgttccgcgag | *zif-1* NS XmaI | pWZ131 |
| oWZ259 | gc **gctagc** ttattgttgaatatttatcg | *zif-1* CAS NheI with stop codon | pWZ131 |
| oWZ316 | aagagcatgtcatacggtgtttaagagctatgctggaaacagc | Sense primer to generate *rab-10* sgRNA #1 plasmid by quick-change | pWZ170 |
| oWZ317 | caatttgaagagcatgtcatagtttaagagctatgctggaaacagc | Sense primer to generate *rab-10* sgRNA #2 plasmid by quick-change | pWZ171 |
| oWZ507 | /5Phos/ caaacatttagatttgcaattcaattatatagggacc | Anti-sense primer to generate *rab-10* sgRNA plasmids by quick-change | pWZ170 and 171 |
| oWZ482 | gc **ggcgcgcc** atggacaaaaaatacagcatcggc | Cas9 NS AscI | pWZ243 |
| oWZ483 | gc **ggtacc** ttaggcgtagtctgggacgtcg | Cas9 CAS KpnI with stop codon | pWZ243 |
| oWZ268 | cg **cccggg**  atgacagaatacaaaacgcgac | *zf1* NS XmaI | pWZ242 |
| oWZ178 | gc **ggtacc**  tttgtatagttcatccatgc | *gfpnovo2* CAS KpnI w/o stop codon | pWZ242 |
