# Supplementary material for: Correction: RAB-10-Dependent Membrane Transport Is Required for Dendrite Arborization
Source: PLoS Genet. 2016 Sep 9;12(9):e1006319. doi: 10.1371/journal.pgen.1006319 (PMC5017700; doi:10.1371/journal.pgen.1006319)
Supplement: S2 File — (PDF) [file pgen.1006319.s002.pdf]

PLOS | GENETICS

RESEARCH ARTICLE

RAB-10-Dependent Membrane Transport Is Required for Dendrite Arborization

Wei Zou[1], Smita Yadav[2], Laura DeVault[2], Yuh Nung Jan[2], David R. Sherwood[1]*

1 Department of Biology, Duke University, Durham, North Carolina, United States of America, **2** Howard Hughes Medical Institute, Department of Physiology, University of California, San Francisco, San Francisco, California, United States of America

* david.sherwood@duke.edu

Abstract

Formation of elaborately branched dendrites is necessary for the proper input and connectivity of many sensory neurons. Previous studies have revealed that dendritic growth relies heavily on ER-to-Golgi transport, Golgi outposts and endocytic recycling. How new membrane and associated cargo is delivered from the secretory and endosomal compartments to sites of active dendritic growth, however, remains unknown. Using a candidate-based genetic screen in *C. elegans*, we have identified the small GTPase RAB-10 as a key regulator of membrane trafficking during dendrite morphogenesis. Loss of *rab-10* severely reduced proximal dendritic arborization in the multi-dendritic PVD neuron. RAB-10 acts cell-autonomously in the PVD neuron and localizes to the Golgi and early endosomes. Loss of function mutations of the exocyst complex components *exoc-8* and *sec-8*, which regulate tethering, docking and fusion of transport vesicles at the plasma membrane, also caused proximal dendritic arborization defects and led to the accumulation of intracellular RAB-10 vesicles. In *rab-10* and *exoc-8* mutants, the trans-membrane proteins DMA-1 and HPO-30, which promote PVD dendrite stabilization and branching, no longer localized strongly to the proximal dendritic membranes and instead were sequestered within intracellular vesicles. Together these results suggest a crucial role for the Rab10 GTPase and the exocyst complex in controlling membrane transport from the secretory and/or endosomal compartments that is required for dendritic growth.

CrossMark
click for updates

OPEN ACCESS

Citation: Zou W, Yadav S, DeVault L, Jan YN, Sherwood DR (2015) RAB-10-Dependent Membrane Transport Is Required for Dendrite Arborization. PLoS Genet 11(9): e1005484. doi:10.1371/journal.pgen.1005484

Editor: Andrew D. Chisholm, University of California San Diego, UNITED STATES

Received: March 9, 2015

Accepted: August 5, 2015

Published: September 22, 2015

Copyright: © 2015 Zou et al. This is an open access article distributed under the terms of the Creative Commons Attribution License, which permits unrestricted use, distribution, and reproduction in any medium, provided the original author and source are credited.

Data Availability Statement: All relevant data are within the paper and its Supporting Information files.

Funding: This work was supported by a Pew Scholars Award, and National Institutes of Health Grant GM079320 to DRS. The funders had no role in the design, data collection and analysis, decision to publish, or preparation of the manuscript.

Competing Interests: The authors have declared that no competing interests exist.

Author Summary

Dendrites are cellular extensions from neurons that gather information from other neurons or cues from the external environment to convey to the nervous system of an organism. Dendrites are often extensively branched, raising the question of how neurons supply plasma membrane and dendrite specific proteins from the source of synthesis inside the cell to developing dendrites. We have examined membrane trafficking in the PVD neuron in the nematode worm *C. elegans* to investigate how new membrane and dendrite proteins are trafficked. The PVD neuron is easy to visualize and has remarkably long and widely branched dendrites positioned along the skin of the worm, which transmits information

about harsh touch and cold temperature to the nervous system. We have discovered that a key organizer of vesicle trafficking, the RAB-10 protein, localizes to membrane vesicles and is required to traffic these vesicles that contain plasma membrane and dendrite proteins to the growing PVD dendrite. Further, our work revealed that a complex of proteins, termed the exocyst, that helps fuse membrane vesicles at the plasma membrane, localizes with RAB-10 and is required for dendrite branching. Together, our work has revealed a novel mechanism for how neurons build dendrites that could be used to help repair damaged neurons in human diseases and during aging.

Introduction

Dendrites and axons are two distinct functional and morphological domains of neurons. Due to the complexity and heterogeneity in the morphology of dendrites, it has been challenging to study the development of dendrites in comparison with the more uniform, simply structured thread-like axons. Previous studies have shown that dendritic growth relies heavily on the secretory pathway and endosomal function[1,2]. In *Drosophila*, loss-of-function mutations in genes encoding the small GTPases Rab1 and Sar1, which are key regulators of ER-to-Golgi vesicular transport [3], severely reduce the growth of dendrites. Notably, loss of Rab1 and Sar1 do not diminish axon outgrowth, suggesting that the mechanisms underlying the extension of dendrites and axon are distinct [1]. Furthermore, Golgi outposts, which are primarily found in dendrites but not axons, play an important role in supplying membranes for dendritic branching and growth [1]. These experiments suggest that membrane components generated in the ER and trafficked to the Golgi are essential for dendritic growth. In addition, Rab5 and Rab11--dependent endocytic membrane trafficking has also been implicated in dendrite morphogenesis [2,4]. The molecular mechanisms that deliver membranes from the Golgi, Golgi-outposts and endosomes to the dendritic plasma membrane, however, are unclear.

To identify the membrane trafficking mechanisms that support dendrite branching and growth, we use the *C. elegans* multi-dendritic PVD neurons as a model. The PVD neurons exist as a pair, PVDL and PVDR, and they function to detect harsh mechanical forces and cold temperatures [5–7]. Each PVD neuron sits on one side of the animal and has a single axon that extends to the ventral nerve cord, as well as a highly branched dendritic arbor that covers most of the body, except for the neck and head [8]. Recently, the transmembrane leucine-rich repeat protein DMA-1 was identified as a PVD dendritic receptor. DMA-1 recognizes skin-derived pre-patterned cues that promote dendrite stabilization and branching [9–11]. In addition, the claudin-like transmembrane protein HPO-30 also promotes dendrite stabilization [12]. Both DMA-1 and HPO-30 are dendrite specific proteins that are rarely observed in axons. The mechanisms that regulate their sorting and trafficking to the dendritic membranes are still unknown [9,12].

The small GTPase Rab10, which is an ortholog to the yeast Sec4p protein that controls post-Golgi vesicle trafficking, has been shown to mediate polarized membrane addition during axonal growth in mammals. In axons Rab10 is activated by the mammalian ortholog of the *Drosophila* gene Lethal giant larvae, Lgl1. The Lgl1 protein dissociates the Rab10-GDI complex [13]. Activated Rab10 then interacts with multiple effector proteins to direct distinct steps of axonal membrane addition. These include an initial interaction with myosin Vb (MYO5B), which controls the biogenesis of post-Golgi Rab10 carriers [14]. Rab10 then binds with c-Jun N-terminal kinase-interacting protein 1 (JIP1) to facilitate anterograde transport of Rab10 cargos [15]. Finally, Rab10 binds myristoylated alanine-rich C-kinase substrate (MARCKS). The

Rab10-MARCKS interaction allows the docking and fusion of Rab10 vesicles with the axonal plasma membrane [16]. Unlike extensive studies on the role of Rab10 in axonal growth, it is unclear whether Rab10 is required for dendrite arborization during development.

Several studies have shown association between the conserved exocyst complex and Rab10 GTPases [17]. The exocyst complex is composed of eight subunits. In *C. elegans* these are encoded by the genes *sec-3*, *sec-5*, *sec-6*, *sec-8*, *sec-10*, *sec-15*, *exoc-7* and *exoc-8* [18]. The exocyst complex functions as the effector of the yeast Rab10 ortholog Sec4p, and facilitates tethering, docking and fusion of secretory vesicles during bud formation [17]. The exocyst complex also associates with Rab10 in renal epithelial cells and may mediate membrane transport to the primary cilium [19]. Both Rab10 and the exocyst complex are further required for the exocytic transport of the glucose transporter Glut4 [20,21]. However, whether Rab10 and the exocyst complex function together during other processes, such as dendritic growth and branching, is not clear.

Here, we report that loss of the *C. elegans rab-10* gene reduces dendritic arborization of the PVD neuron. We show that RAB-10 functions cell-autonomously, and localizes to the Golgi and the early endosomes in the PVD neurons. Further, we find that deficiencies in *rab-10* and the exocyst subunits cause accumulation of the dendritic membrane proteins DMA-1 and HPO-30 within intracellular vesicles. We also show that Rab10 and the exocyst complex are required for dendrite arborization in *Drosophila*, and dendritic spine formation in mammalian neurons. Together, these data suggest that Rab10 and the exocyst complex play a conserved

Table 1. PVD dendrite morphogenesis is defective in *rab-10*, *rab-1* and exocyst mutants.

Genotype	Human homologue	% of animals with a PVD dendrite arborization defect[2]
Wild-type	N/A	0 (n = 60)
rab-10(ok1494)	RAB10	100 (n = 66)
rab-10(dx2)	RAB10	90 (n = 50)
Ex[ser2p3>rab-1 DN] line 1	RAB1A	53 (n = 51)
Ex[ser2p3>rab-1 DN] line 2	RAB1A	46 (n = 54)
exoc-8(ok2523)	EXOC8	100 (n = 50)
sec-8(ok2187,m+z-)[1]	EXOC4	100 (n = 50)
exoc-7(ok2006)	EXOC7	0 (n = 50)
sec-10(tm3437, m+z-)[1]	EXOC5	8 (n = 50)
sec-5(tm1443, m+z-)[1]	EXOC2	0 (n = 52)
rab-8(tm2526)	RAB8B	2 (n = 50)
ehbp-1(ok2141)[1]	EHBP1	0 (n = 55)
Ex[ser2p3>rab-5 DN] line 1	RAB5B	0 (n = 58)
Ex[ser2p3>rab-5 DN] line 2	RAB5B	0 (n = 55)
Ex[ser2p3>rab-11.1 DN] line 1	RAB11A	0 (n = 55)
Ex[ser2p3>rab-11.1 DN] line 2	RAB11A	0 (n = 53)
rme-1(b1045)	EHD1	0 (n = 60)
chat-1 (ok1681)[1]	TMEM30A	2 (n = 50)

[1]These strains were maintained as heterozygotes using balancers and homozygous progeny derived from heterozygous mothers were examined.

[2]An animal was considered as abnormal if it lacked more than four menorahs (either by lacking quaternary dendrites, tertiary dendrites or secondary dendrites) or if it showed truncated primary dendrites.

doi:10.1371/journal.pgen.1005484.t001

role in controlling Golgi-to-plasma membrane and/or endosome-to plasma membrane trafficking required for dendrite morphogenesis.

Results

Loss of *rab-10* affects PVD dendritic arborization

To identify potential regulators of membrane trafficking during dendritic branching and growth of the multi-dendritic PVD neuron, a candidate-based genetic screen was performed. We crossed animals harboring mutations in genes encoding proteins important for various membrane trafficking pathways with a PVD neuron-specific fluorescent marker (*F49H12.4>gfp* or *ser2prom3>gfp*). The morphology of PVD dendritic arbors was then examined. We found that two putative null alleles of *rab-10* presented severely abnormal PVD dendrite morphology (Table 1 and Fig 1A–1E). In the proximal region (including the middle and tail areas), *rab-10* (*ok1494)* and *rab-10* (*dx2*) mutant animals contained far fewer dendritic branches compared to wild-type. For example, a count of secondary dendrites within a 100μm long region along the primary dendrite anterior to the PVD cell body in *rab-10* (*ok1494*) and *rab-10* (*dx2*) animals revealed an average of 1.1±0.3 and 2.8±0.7 secondary dendrites. In comparison, wild-type animals within this region contained 11.1±0.5 secondary dendrites (Fig 1F and 1G). Tertiary and quaternary branches were even more affected and were essentially absent in the proximal region of *rab-10* (*ok1494*) and *rab-10* (*dx2*) animals (Fig 1F, 1H and 1I). Interestingly, in the distal area of the PVD, the dendritic branching and growth were minimally affected by loss of *rab-10*, indicating that a *rab-10*-independent mechanism mediates distal dendritic branching (Fig 1A–1E, 1G–1I). The growth of the primary dendrite and axon of the PVD appeared normal in *rab-10* deficient animals (Fig 1A–1E, and S1 Fig). *rab-10* was also required for the dendritic arborization of the FLP neuron, which covers the head region and has a similar morphology and function as the PVD neuron (S2 Fig) [8]. However, *rab-10* was dispensable for the growth of unbranched dendrites of OLL, AWB, and AWC neurons, suggesting a specific role of *rab-10* in mediating the growth of dendritic branches in multi-dendritic neurons (S2 Fig). Together, these data indicate that RAB-10 is required for the elaboration of branched dendrites in *C. elegans*.

RAB-10 functions cell-autonomously in the PVD neuron

To determine if RAB-10 activity is required within the PVD, we tagged full length RAB-10 at its N-terminus with GFP and expressed this construct under a PVD-specific promoter (*ser2-prom3*) in *rab-10(ok194)* animals. GFP::RAB-10 expressed from multicopy extrachromosomal arrays in the PVD neuron fully rescued the morphology of the PVD dendritic arbor in most animals in two independent lines (Fig 2A–2C), indicating that RAB-10 functions within the PVD to promote proximal dendritic arborization. As a GTPase, RAB-10 cycles between the GDP-bound inactive form and GTP-bound active form. To test whether its function in promoting dendrite branching and growth requires GTPase activity, we expressed both GDP-locked (T23N) and GTP-locked (Q68L) forms of RAB-10 and examined their rescuing ability. Dominant-negative RAB-10 (T23N) not only failed to rescue the proximal PVD defects in *rab-10* (*ok1494*) mutants, but also disrupted the distal dendrite arbor in wild-type animals (Fig 2C, S3 Fig). In contrast, constitutively active RAB-10 (Q68L) fully rescued the PVD dendrite morphogenesis defects in *rab-10* (*ok1494*) mutant animals (Fig 2C). Over-expressing constitutively active RAB-10 (Q68L) did not cause over-growth of dendrites in PVD neuron (S4 Fig), suggesting that other factors limit dendrite growth and patterning. We conclude that RAB-10 functions cell autonomously in the PVD neuron and requires GTPase activity to promote PVD dendrite morphogenesis.

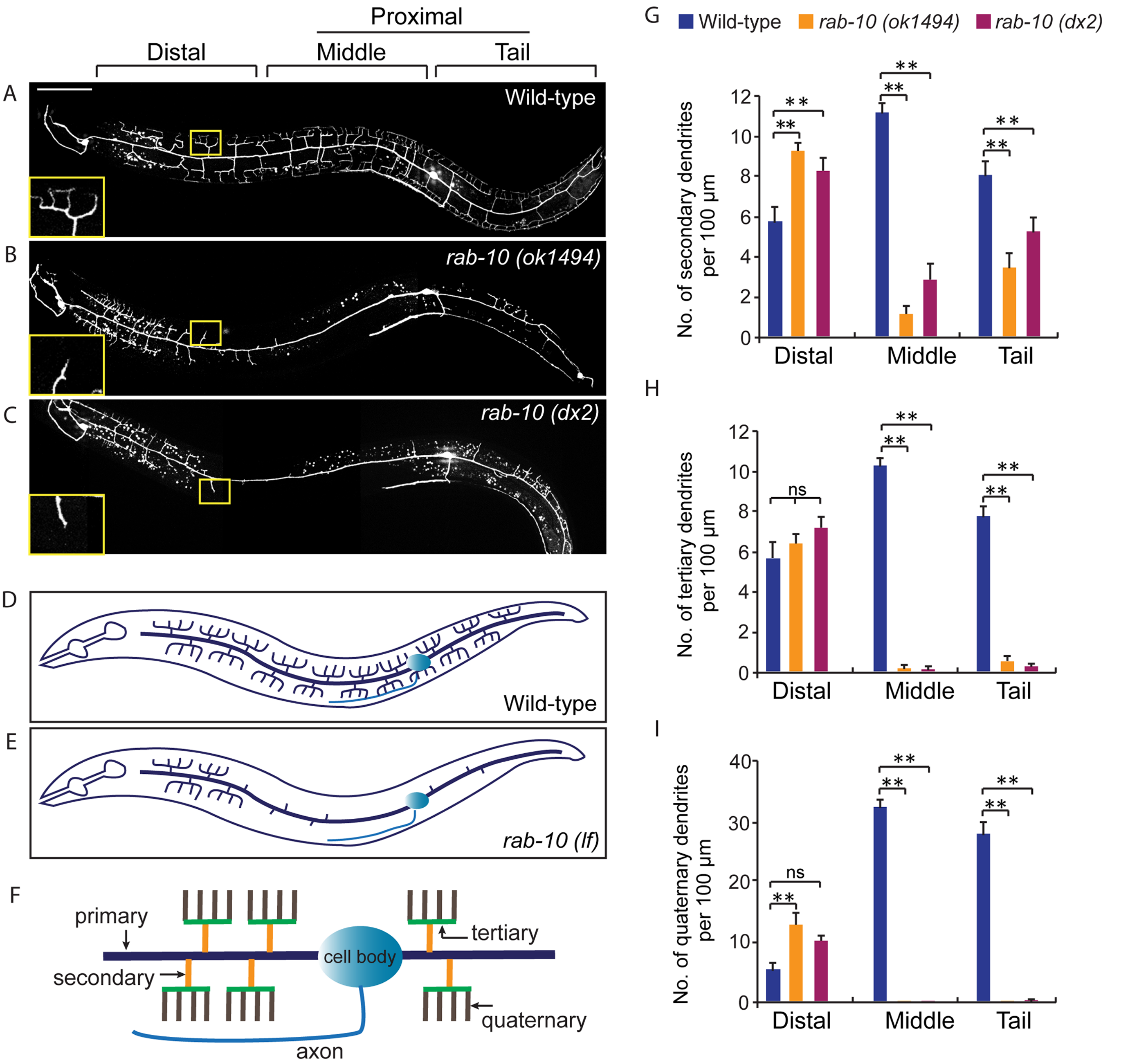

Fig 1. RAB-10 promotes formation of proximal PVD dendritic arbors. (A-C) Images show PVD morphology of (A) wild-type, (B) *rab-10* (*ok1494*), and (C) *rab-10* (*dx2*) mutant worms at the L4 stage. PVD morphology was visualized using a PVD>GFP reporter line *wdIs51* (*F49H12.4>gfp*). The inset images are enlarged views (2.5 fold) of the regions indicated by the boxes. All images are maximum intensity projections of z-stacks of images acquired using a spinning disk confocal microscope. (D, E) A schematic image of PVD morphology in (D) wild-type and (E) *rab-10* loss-of-function mutant worms. (F) A schematic image of menorahs in the PVD neuron. (G, H) Quantification of number of (G) secondary dendrites, (H) tertiary dendrites and (I) quaternary dendrites in three 100 μm x 100 μm areas from the distal, middle and tail region, respectively. 10 animals at the L4 stage were randomly selected and quantified for each genotype. Scale bar, 50 μm. A one-way ANOVA followed by post-hoc comparisons using the Dunnett's test was used to compare wild-type and mutant animals. ns: not significant; **: P<0.01. Error bars report ±SEM.

doi:10.1371/journal.pgen.1005484.g001

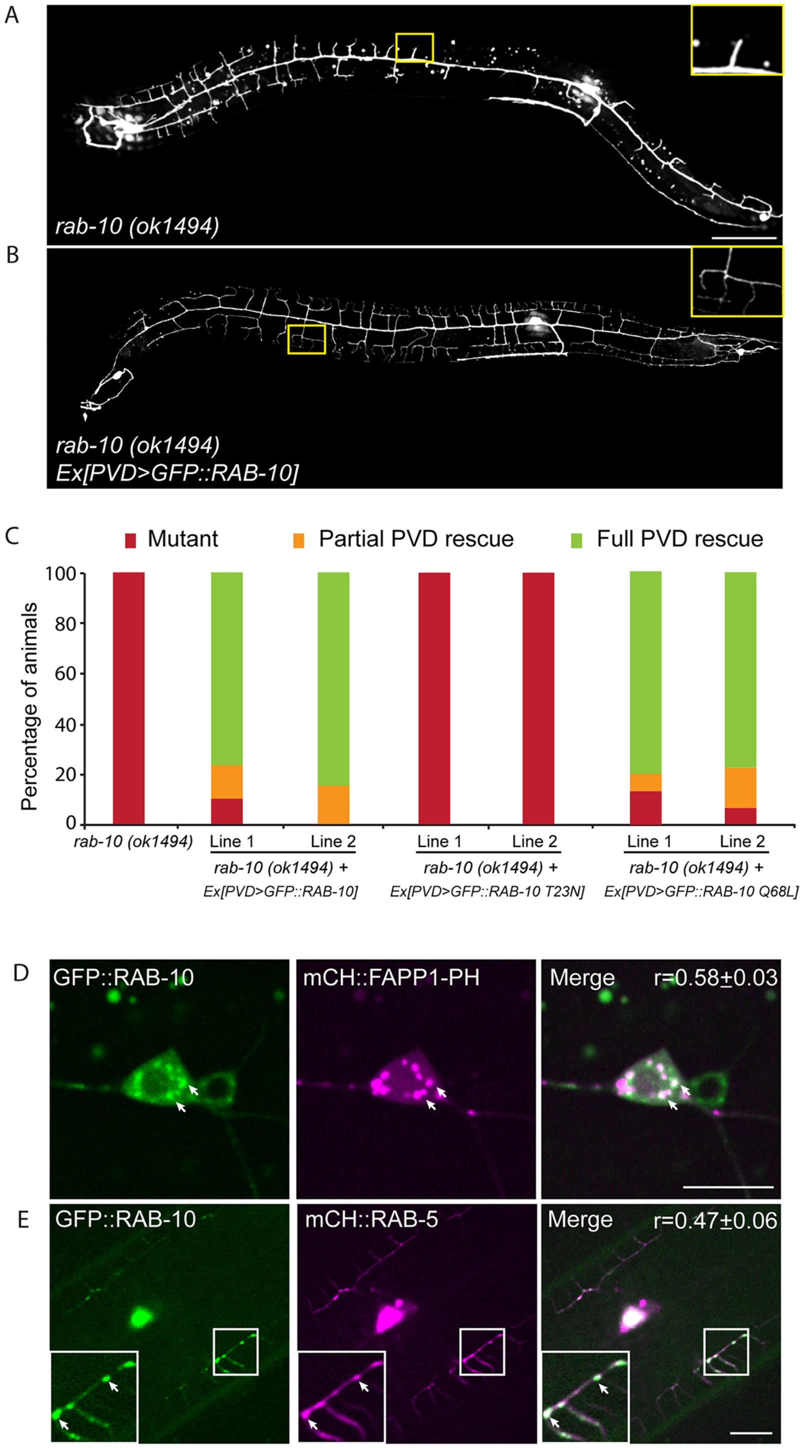

Fig 2. RAB-10 functions cell-autonomously in the PVD neuron. (A, B) The dendritic morphogenesis defect in *rab-10* (*ok1494*) mutant animals was rescued by PVD-specific expression of GFP::RAB-10 driven from a multicopy extrachromosomal array. PVD morphology was visualized using the PVD>*gfp* marker strain *wdIs51*. The inset images are enlarged views (2.5 fold) of the regions indicated by the boxes. Both images are maximum intensity projections of z-stacks. Scale bar, 50 μm. (C) Quantification of the cell-autonomous rescue of dendritic morphogenesis defect by cell-specific expression of wild type, GDP-locked, and GTP-locked forms of RAB-10. For each construct, at least two *rab-10* (*ok1494*) lines carrying arrays expressing *ser2prom3*> *gfp*::*rab-10* WT, GDP-locked (*T23N*), and GTP-locked (*Q68L*) forms were generated. Animals were categorized based on their PVD dendrite morphology. At least 29 animals at the L4 or young adult stage

were scored for each line. Partial PVD rescued animals contained one or more full menorahs in the proximal region. Full PVD rescued animals contained PVD dendritic arbors that were indistinguishable from wild type animals. (D) Colocalization of GFP::RAB-10 and mCHERRY::FAPP1-PH in the PVD. Magenta, mCHERRY::FAPP1-PH; green, GFP::RAB-10. (E) Colocalization between GFP::RAB-10 and mCHERRY::RAB-5 in PVD. Magenta, mCHERRY::RAB-5; green, GFP::RAB-10. r-value reports mean Pearson's correlation coefficient ± SEM for colocalization of GFP::RAB-10 and mCHERRY::FAPP1-PH, and GFP::RAB-10 and mCHERRY::RAB-5. At least 15 animals at the L4 or young adult stage were quantified for each group of colocalization analysis. The inset images are enlarged views (2 fold) of the regions indicated by the boxes. Arrows indicate vesicles co-labeled by both GFP and mCherry reporters. Error bars report ±SEM. Scale bar, 10μm.

doi:10.1371/journal.pgen.1005484.g002

RAB-10 colocalizes with the Golgi and endosomes in the PVD neuron

RAB-10 orthologs are known to regulate Golgi to plasma membrane vesicle trafficking as well as endocytic recycling events [22–26]. To determine if RAB-10 localizes with either Golgi or endosomes in the PVD neuron we tagged full length RAB-10 with GFPnovo2, a mutant form of GFP that is brighter. We generated a single copy insertion line using *miniMos* method to minimize potential ectopic localization of RAB-10 protein induced by over-expression [27,28]. Similar to the multicopy line, we crossed this line into the *rab-10* null mutant and found that it fully rescued the PVD dendrite arborization defect (S5 Fig). Many intracellular vesicles were labeled by GFP::RAB-10 in the PVD dendrites. Notably, there was a strong correlation between GFP::RAB-10 localization and mCherry::FAPP1-PH (Golgi reporter) and a strong correlation of GFP::RAB-10 and mCherry::RAB-5 (early endosome reporter) (Fig 2D and 2E) [29]. These observations are consistent with previous studies showing RAB-10 localization to the Golgi and early endosomes in the *C*. *elegans* intestinal cells [24]. Supporting a role in mediating vesicular trafficking, time-lapse recordings revealed that GFP::RAB-10 labeled vesicles moved bidirectionally along dendrites, consistent with localization to transport vesicles (S1 Movie). Together, these data suggest that RAB-10 might mediate PVD dendrite outgrowth by regulating Golgi-to-membrane trafficking or endosomal membrane recycling events.

To attempt to determine whether the PVD dendrite morphogenesis defect in *rab-10* mutants might be due to a role for RAB-10 in Golgi-to-plasma membrane trafficking, endocytic recycling, or both, we perturbed each pathway and tested whether it altered PVD dendritic arborization. Consistent with previous studies in *Drosophila* and rat hippocampus neurons implicating ER-to-Golgi trafficking in dendritic growth, over-expressing dominant-negative RAB-1 (a key regulator of ER-to-Golgi trafficking) caused dramatically reduced branching in the PVD (Table 1, and S6 Fig) [1]. In contrast, genetic loss of *rme-1* or *chat-1*, and over-expression of dominant-negative RAB-5 or RAB-11.1 (all key regulators of endocytic recycling) [30–33], had no effect of PVD dendrite morphology (Table 1). Together, these data suggest a role for RAB-10 in mediating Golgi-to-plasma membrane trafficking that is important for dendrite morphogenesis. Notably, however, we cannot rule the possibility that RAB-10 regulates endosomal recycling independent of *rme-1*, *rab-5* or *rab-11.1*. Thus, RAB-10 might also regulate an endosome-to-plasma membrane transport pathway independent of RME-1, RAB-5, RAB-11.1 function in the PVD neuron that is required for dendritic arborization.

Loss of exocyst complex components cause defects in PVD dendritic morphogenesis

Rab GTPases are molecular switches that exert their functions by recruiting and releasing specific effectors [34]. We next sought to identify possible effectors that function with RAB-10 in mediating the branching and growth of PVD dendrites. We first examined EHBP-1, an Eps 15 domain binding protein that acts as an effector of RAB-10 in endocytic recycling and secretory

pathways [26]. Mutant animals of *ehbp-1*, however, had normal PVD dendritic arbors (Table 1). Importantly, we cannot fully exclude the possibility that the PVD dendrite development was rescued by maternally loaded *ehbp-1* mRNA or EHBP-1 protein, since we can only examine *ehbp-1* homozygous animals derived from heterozygous mothers. We next tested members of the exocyst complex, an established effector of yeast Sec4p with which RAB-10 shares high homology [35]. Notably, loss of the exocyst subunit *exoc-8* in viable null mutant animals or loss of *sec-8* in the mutant progeny of heterozygous *sec-8* mutant mothers, caused a PVD dendrite morphogenesis defect in the proximal but not in the distal region (Table 1, and Fig 3A–3F)[36]. Further, the growth of the primary dendrite and the axon was normal (Fig 3A–3C, S1 Fig). This phenotype was similar to *rab-10* mutants, although the growth of secondary dendrites was only mildly affected in *exoc-8* and *sec-8* mutant worms (Fig 3A–3F). Expression of *exoc-8* cDNA under the PVD-specific promoter (*ser2prom3*) fully rescued the dendritic arborization defect in *exoc-8* (*ok2523*) mutant worms, indicating that the exocyst complex functions in the PVD to promote dendritic arborization (Fig 3G).

Homozygous mutants of two other exocyst components, *sec-5* and *sec-10*, derived from the heterozygous mothers did not show any obvious PVD dendrite defect (Table 1). To test whether this was due to maternal rescue, we used a newly developed targeted protein degradation system to specifically remove the SEC-5 protein from the PVD [37]. This system takes advantage of cell type specific expression of the E3 ubiquitin ligase substrate-recognition subunit ZIF-1, which recognizes proteins tagged with the 36 amino acid ZF1 zinc-finger domain. To selectively remove SEC-5 in the PVD neuron, we drove ZIF-1 in PVD using the *ser2prom3* promoter in a *sec-5*::*zf1*::*yfp* knock-in strain (*sec-5(xn51))*—a strain where both copies of the endogenous *sec-5* genes are tagged with *zf1* (Fig 4A) [37]. Confirming a role for the exocyst complex in promoting dendritic arborization, depleting ZF1::YFP tagged SEC-5 in PVD resulted in PVD dendrite arborization defects (Fig 4C–4E). In some of these animals, both distal and proximal regions lacked menorah structures, suggesting that *sec-5* might be important for the growth of both proximal and distal dendrites (Fig 4D). Control animals expressing *ser2-prom3*>ZIF-1 transgene alone and control animals expressing ZF1::YFP tagged SEC-5 alone had normal PVD dendrite morphology (Fig 4B and 4E). We conclude that the exocyst complex plays a cell-autonomous role in promoting dendritic arborization and that maternal contributions of some of exocyst components contribute to this function. Further, based on the more severe PVD phenotype after ZF-1 tag-directed loss of *sec-5* versus the *exoc-8* null mutant, our results also suggest that exocyst components have different requirements (perhaps reflecting their differential necessity for exocyst activity) during PVD dendrite morphogenesis.

RAB-10 and EXOC-8 mediate transport of the dendritic membrane proteins DMA-1 and HPO-30

Yeast Sec4p and the exocyst complex function together to promote docking and possible fusion of post-Golgi vesicles [17]. Thus, we hypothesized that the PVD dendritic arborization defects in *rab-10*, *exoc-8* and *sec-8* mutants might in part be due to the failure of docking of post-Golgi vesicles. To test this, we built fluorescent reporters for the dendritic membrane proteins, DMA-1 and HPO-30, transmembrane proteins expressed in PVD neurons that function to mediate dendritic branching and stabilization [9–12]. Consistent with previous studies, DMA-1::GFP and HPO-30::GFP were localized in the dendritic membranes, and in some intracellular vesicles in wild-type animals (Fig 5A–5C, and S7 Fig) [9,12]. These two transgene reporters likely represent endogenous protein localization, as the transgenes rescued the PVD dendrite arborization defects of *dma-1*(*tm5159*) and *hpo-30*(*ok2147*) mutants (S5 Fig).

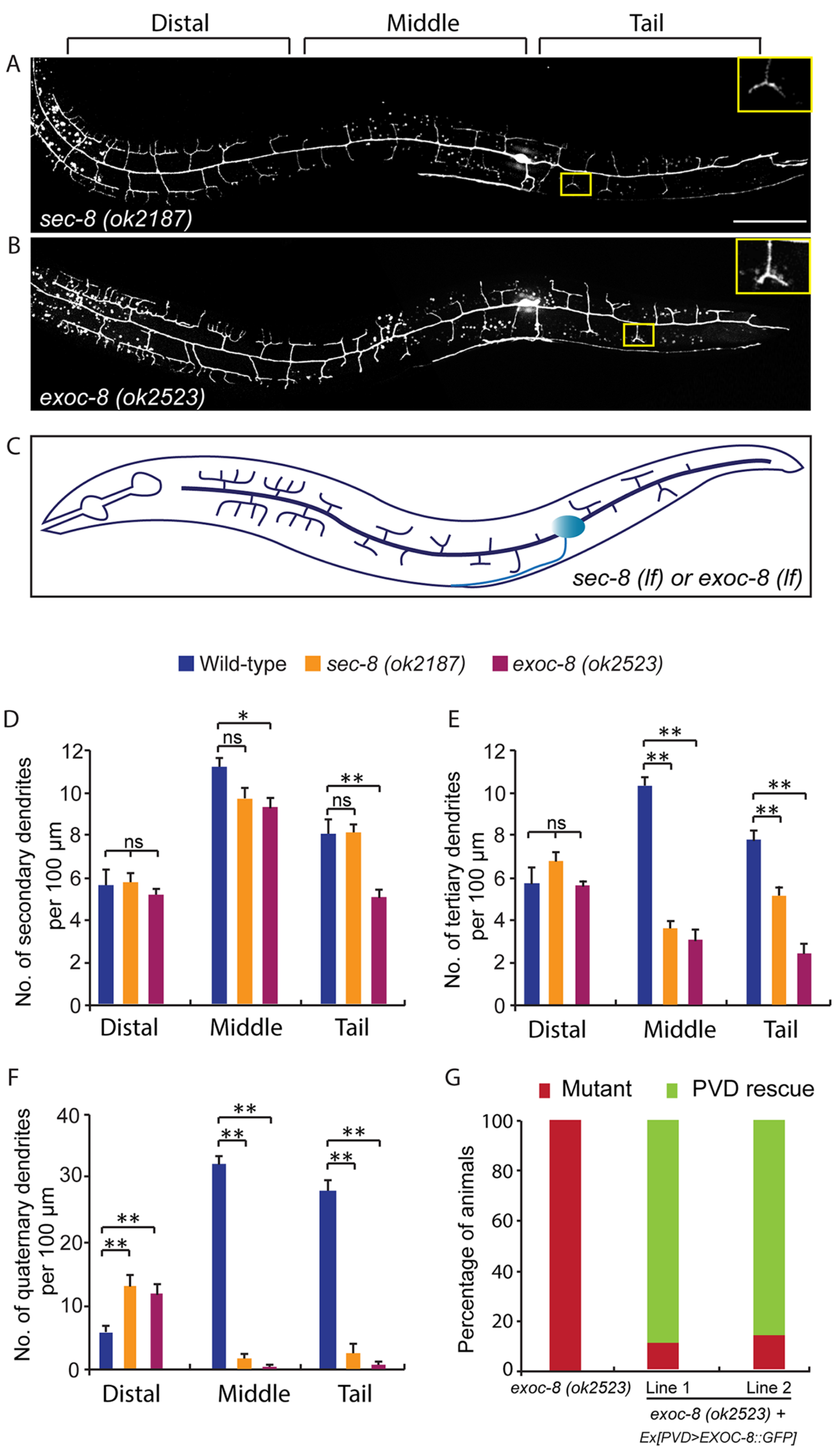

Fig 3. Mutations in *exoc-8* and *sec-8* reduce PVD dendritic arborization. (A, B) Images show PVD morphology of (A) *sec-8* (*ok2187*) and (B) *exoc-8* (*ok2523*) mutant animals at the L4 stage. PVD morphology was visualized using the *wdIs51* strain. The inset images are enlarged views (2.5 fold) of the regions indicated by the boxes. Both images are maximum intensity projections of z-stacks. Scale bar, 50 μm. (C) A schematic of PVD morphology of *sec-8* (*ok2187*) and *exoc-8* (*ok2523*) mutant animals. (D-F) Quantification of the number of (D) secondary dendrites (E) tertiary dendrites and (F) quaternary dendrites in three 100 μm x 100 μm areas from the distal, middle and tail regions, respectively. 10 animals at the L4 stage were quantified for each genotype. A one-way ANOVA followed by post-hoc comparisons using the Dunnett's test was used to compare wild-type and mutant animals. ns: not significant; *: P<0.05; **: P<0.01. (G) Quantification of cell-

autonomous rescue of dendritic morphogenesis defect by cell-specific expression of *exoc-8*::*gfp* construct. Results of two independent lines are shown. At least 38 animals at the L4 or young adult stage were scored for each line.

doi:10.1371/journal.pgen.1005484.g003

Consistent with a role in docking and fusion of vesicles, loss of *rab-10* and *exoc-8* caused severe accumulation of DMA-1::GFP containing vesicles within the growing PVD dendrites (Fig 5D–5I). We quantified the number of vesicular units (which might be a single vesicle or a vesicle cluster) in three areas—the distal, middle and proximal regions. We found that wild-type animals contained on average 29.6±1.9 vesicular units in a 76.8μm x 76.8 μm area from the middle region of the PVD. In contrast *rab-10* (*ok1494*) and *exoc-8* (*ok2523*) mutant worms contained a dramatic two-to-three fold increase in vesicular units—65.6±2.8 and 87.8±6.8, respectively (Fig 5J). Furthermore, in wild-type animals the DMA-1::GFP containing vesicles mainly localized to the primary dendrites, and rarely appeared in the higher-order dendrites (including the secondary, tertiary and quaternary dendrites; Fig 5A–5C). In contrast, in *rab-10* (*ok1494*) and *exoc-8* (*ok2523*) mutant worms, numerous vesicles appeared in the higher-order dendrites (Fig 5D–5I and S8 Fig).

The presence of numerous DMA-1::GFP intracellular vesicles in *rab-10* and *exoc-8* mutants suggested that delivery of DMA-1 to the membrane might be reduced. To test this idea, the fluorescence intensity of DMA-1::GFP at the surface (which we presume is predominantly plasma membrane localization) of the primary dendrites was determined. In the distal region of the dendritic arbor, the intensity of DMA-1::GFP at the primary dendrite in *rab-10* (*ok1494*) and *exoc-8* (*ok2523*) mutant worms was decreased by 63.9% and 47.8% compared with wild-type animals (Fig 5K). In the *rab-10* mutants, reduction of *dma-1* by RNAi-mediated knock-down further suppressed distal dendritic arborization, suggesting that the reduced levels of DMA-1 on the surface of distal dendrites is sufficient to promote dendritic stabilization and branching (S9 Fig). In the proximal region, the decrease in DMA-1 was more dramatic. The intensity of DMA-1::GFP at the surface of the primary dendrite in *rab-10* (*ok1494*) and *exoc-8* (*ok2523*) mutant worms was decreased by 89.5% and 90.3% in the middle regions, and 94.4% and 94.8% in the tail regions compared to wild-type animals (Fig 5K). HPO-30::GFP showed similar vesicular accumulation and decreased dendrite surface localization in *rab-10* (*ok1494*) and *exoc-8* (*ok2523*) mutant animals (S7 Fig). These results offer compelling evidence that *rab-10* and exocyst activity are required for the vesicular delivery of DMA-1 and HPO-3 to the dendritic membrane.

EXOC-8 and SEC-8 promote fusion of RAB-10 vesicles

The similar phenotypes after loss of exocyst components and *rab-10*, as well as functions of these molecules in yeast and vertebrates in delivering vesicles to the plasma membrane [17,19,35,38,39] led us to examine whether RAB-10 and exocyst components coexist in vesicles in the PVD dendrites. EXOC-8::GFP and mCherry::RAB-10 strongly colocalized on intracellular vesicles (Fig 6A), indicating that the exocyst might function together with *rab-10* to mediate vesicle delivery. Next, we examined the subcellular localization of RAB-10 in animals harboring mutations in the exocyst components *exoc-8* and *sec-8*. Loss of these exocyst components caused a dramatic accumulation of RAB-10 labeled vesicles in the PVD dendrites (Fig 6B). Wild-type animals contained 39.8±2.6 GFP::RAB-10 vesicular units in a 88.1μm x 88.1 μm area from the middle region of the PVD neuron. Worms with mutations in *exoc-8* (*ok2523*) and *sec-8* (*ok2187*) contained over three fold more GFP::RAB-10 vesicular unites—129.7±7.5 and 127.6±2.7, respectively (Fig 6C). To determine whether the accumulated vesicles containing GFP::RAB-10 were the same population observed in *exoc-8*(*ok2523*) mutants carrying

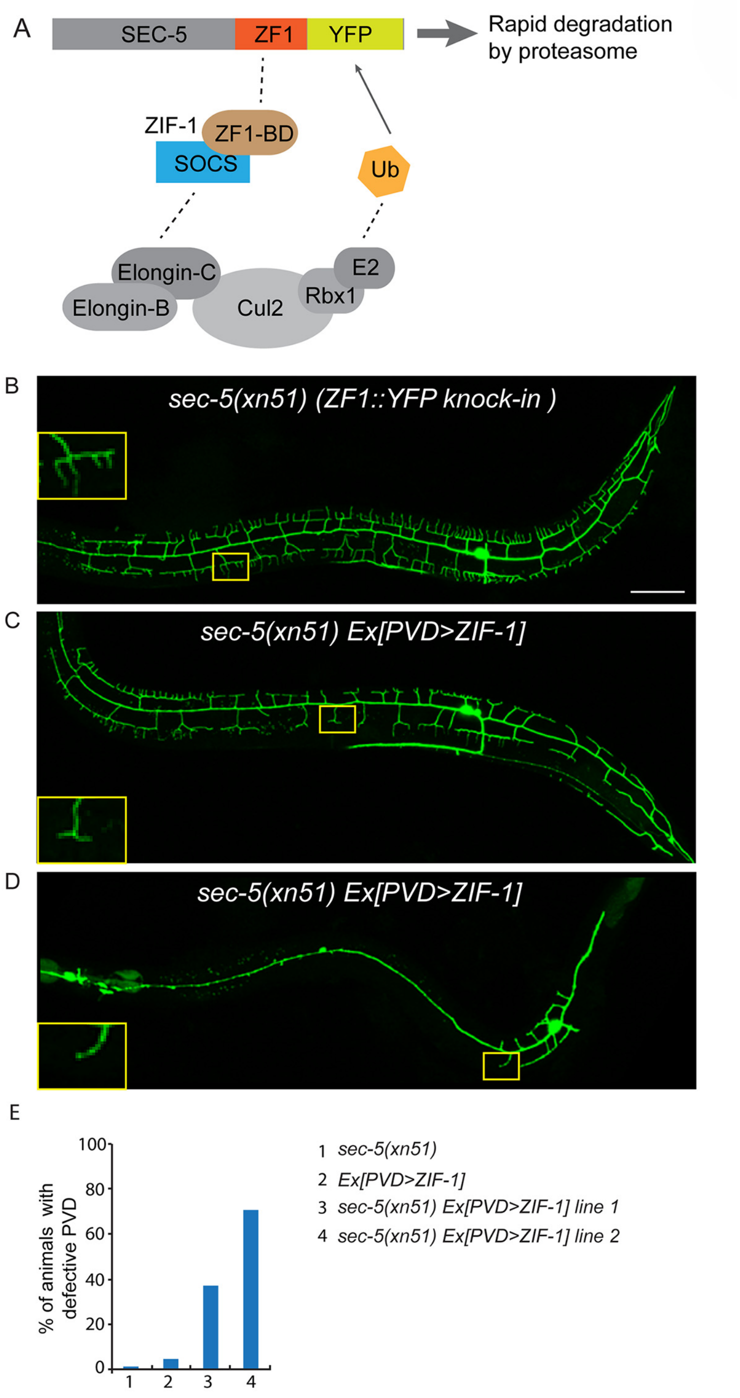

Fig 4. ZIF-1-mediated degradation of SEC-5 in the PVD reduces dendrite arborization. (A) A cartoon showing how ZIF-1 recruits the SEC-5::ZF1::YFP fusion protein to an ECS E3 ligase complex for ubiquitylation (Ub) and subsequent rapid degradation by the proteasome. (B-D) Fluorescence images show the morphology of the PVD neuron using the PVD>*myr-gfp* marker strain *wyIs592* in (A) *sec-5*(*xn51*) (expresses SEC-5 tagged with YFP and the ZF1 zinc-finger domain), (B and C) *sec-5(xn51)* with a transgene expressing *ser2prom3>zif-1* (which encodes a E3 ubiquitin ligase substrate-recognition subunit). The inset images are enlarged views (2.5 fold) of the regions indicated by the boxes. All images are maximum intensity projections of z-stacks. L4 or young adult stage animals were examined. Scale bar, 50 μm. (E) Quantification of percentage of animals showing PVD dendrite arborization defect. At least 50 animals at the L4 or young adult stage were quantified for each genotype.

doi:10.1371/journal.pgen.1005484.g004

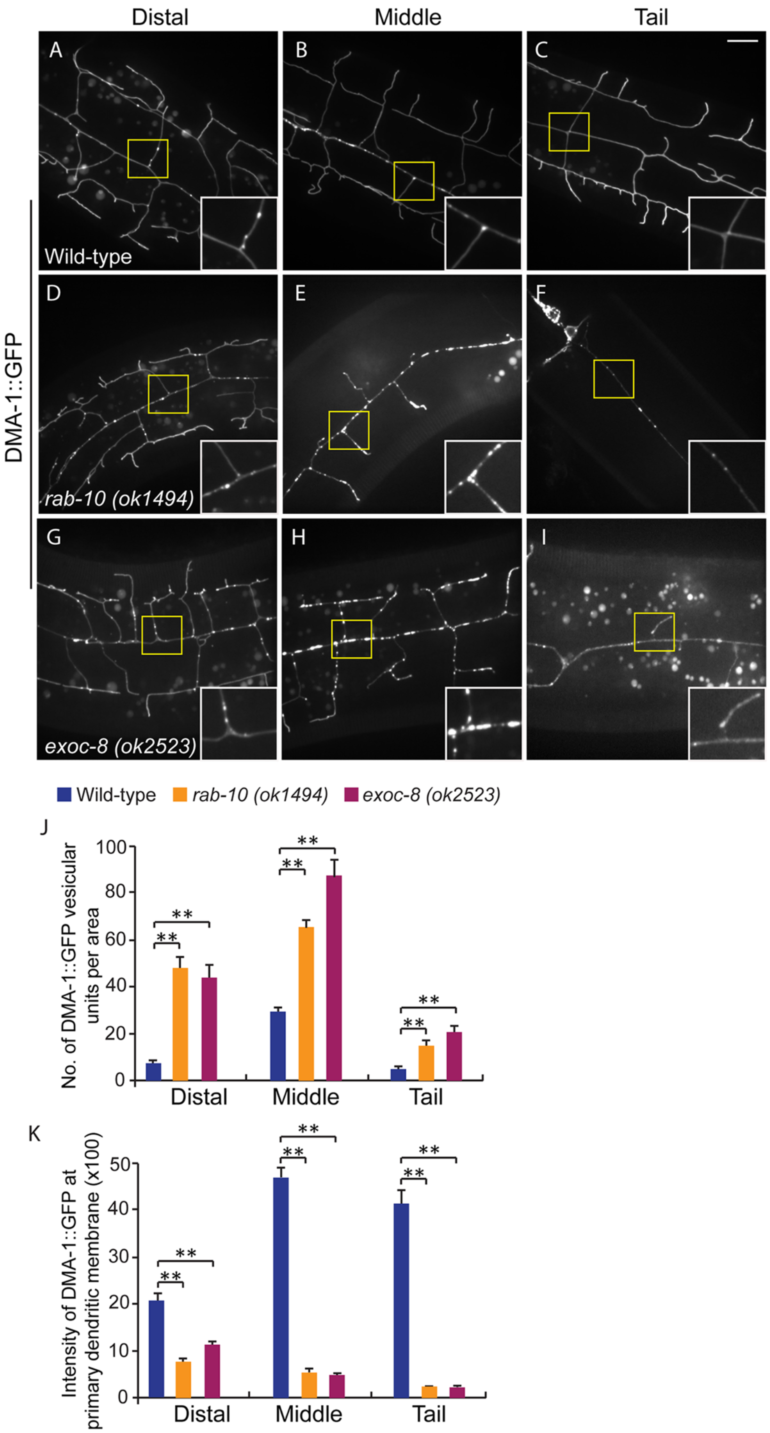

Fig 5. Loss of *rab-10* and *exoc-8* perturbs DMA-1 transport to dendritic membranes. (A-I) Images show subcellular localization of DMA-1::GFP in (A-C) wild-type, (D-F) *rab-10* (*ok1494*) and (G-I) *exoc-8* (*ok2523*) L4 stage animals in the distal, middle and tail region, respectively. All images are maximum intensity projections of z-stacks. Insets are 2.5 fold enlarged views. Scale bars, 10 μm. (J) Quantification of number of vesicles or vesicle clusters per area (76.8μm x 76.8μm) in wild-type, *rab-10* (*ok1494*) and *exoc-8* (*ok2523*) mutant worms in the distal, middle, and tail regions, respectively. At least 8 animals were quantified for each genotype. (K) Quantification of the intensity of DMA-1::GFP at the surface of the primary dendrites in wild-type, *rab-10* (*ok1494*) and *exoc-8* (*ok2523*) mutant worms in the distal, middle, and tail regions, respectively. 10 animals were quantified for each genotype. A one-way ANOVA followed by post-hoc comparisons using the Dunnett's test was used to compare wild-type and mutant animals. **: P<0.01. Error bars report ±SEM.

doi:10.1371/journal.pgen.1005484.g005

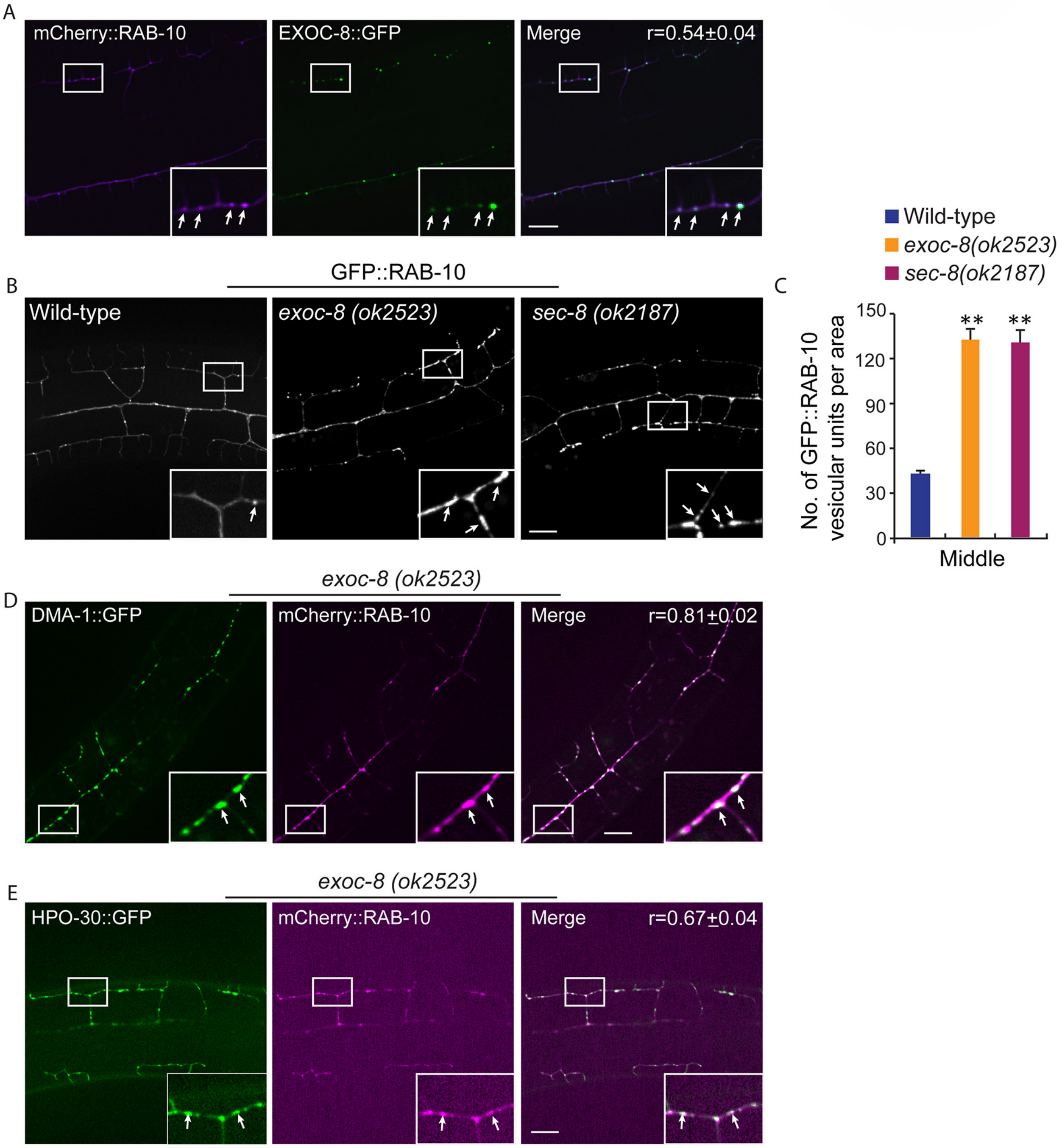

Fig 6. *exoc-8* and *sec-8* are required for fusion of RAB-10 vesicles. (A) mCherry::RAB-10 colocalizes with EXOC-8::GFP. r-value reports the mean Pearson's correlation coefficient ± SEM for colocalization of RAB-10 and EXOC-8. 15 animals at the L4 stage were quantified for each genotype. Insets are 2.5 fold enlarged views. Arrows indicate vesicles co-labeled by both EXOC-8::GFP and mCherry::RAB-10. Scale bar, 10 μm. (B) Images showing GFP:: RAB-10 reporter in wild-type, *exoc-8*(*ok2523*) and *sec-8*(*ok2187*) animals. All images are maximum intensity projections of z-stacks. Insets are 2.5 fold enlarged views. Arrows indicate vesicles labeled by GFP::RAB-10. Scale bar, 10 μm. (C) Quantification of the number of GFP::RAB-10 vesicles or vesicle clusters per area (88.1μm x 88.1μm) in wild-type, *exoc-8*(*ok2523*) and *sec-8*(*ok2187*) animals. 15 animals at the L4 stage were quantified for each genotype. A one-way ANOVA followed by post-hoc comparisons using the Dunnett's test was used to compare wild-type and mutant animals. **: P<0.01. (D) mCherry::RAB-10 colocalizes with DMA-1::GFP in *exoc-8(ok2523)* animals. (E) mCherry::RAB-10 colocalizes with HPO-30::GFP in *exoc-8(ok2523)* animals. r-value reports the mean Pearson's correlation coefficient ± SEM for colocalization of RAB-10 and DMA-1 or HPO-30. 15 animals at the L4 to young adult stage were quantified for each genotype. Insets are 2.5 fold enlarged views. Arrows indicate vesicles co-labeled by both mCherry::RAB-10 and DMA-1::GFP or HPO-30::GFP. Error bars report ±SEM. Scale bar, 10 μm.

doi:10.1371/journal.pgen.1005484.g006

DMA-1::GFP or HPO-30::GFP, we expressed DMA-1::GFP or HPO-30::GFP and mCherry::RAB-10 in *exoc-8*(*ok2523*) mutant animals. Confirming these are the same vesicle population, most of the mCherry::RAB-10 labeled vesicles also contained DMA-1::GFP or HPO-30::GFP (Fig 6D and 6E). To test whether RAB-10 functions to recruit EXOC-8 onto vesicles, the EXOC-8::GFP reporter was crossed into the *rab-10* loss-of-function mutant. EXOC-8::GFP, however still localized to vesicles in PVD neurons in *rab-10* mutants (S10 Fig). Taken together, these results suggest the exocyst complex promotes fusion of RAB-10 carriers within the PVD neuron to facilitate dendritic growth and stabilization, but that the exocyst complex is recruited to these vesicles in a RAB-10 independent manner.

Drosophila and mammalian Rab10 and exocyst subunits mediate dendritic morphogenesis and dendritic spine formation, respectively

To investigate whether Rab10 and exocyst complex-mediated dendritic membrane transport is required for the dendrite morphogenesis in other organisms, we examined *Drosophila* class IV dendritic arborization neurons and cultured rat hippocampal neurons after loss of *rab10* and exocyst components. In *Drosophila*, RNAi mediated knock-down of *rab10* and *exo84* significantly reduced dendrite branching and growth (S11 Fig). Compared to animals treated with control RNAi, *rab10* and *exo84* RNAi targeted loss resulted in a 20% decrease in both the number of total end points and the total dendritic arbor (S11 Fig). ShRNA mediated knock-down of *rab10*, *sec8* and *exoc84* did not alter the total dendrite length in cultured rat embryonic hippocampal neurons, but did lead to a dramatic 67% reduction in the density of dendritic spines at 21 days *in vitro* (DIV) compared to control shRNA treated neurons (S12 Fig). Collectively, we conclude that *rab10* and the exocyst complex have important and likely conserved roles during dendrite morphogenesis.

RAB-8 functions redundantly with RAB-10 to promote dendritic branching

Since some higher-order dendrites in the PVD still formed in *rab-10* null alleles, we hypothesized that another Rab protein might regulate dendritic branching. To test this idea, we examined the function of the RAB-10 related GTPase RAB-8 [40]. Notably, homozygous viable *rab-8(tm2526)* null mutant animals showed normal PVD morphology (Fig 7A). This suggested that if RAB-8 functions in the PVD, it might act redundantly with RAB-10. To test this idea, we first attempted to create animals with null mutations in both *rab-8* and *rab-10*. The *rab-8* and *rab-10* double mutant animals, however, were sterile, which made it challenging to determine PVD morphology [26]. Interestingly, we found that expression of a dominant-negative RAB-8 (T22N) in the PVD neuron caused a severe dendrite morphogenesis defect (S3 Fig). In 15% (n = 40) and 12% (n = 50) of transgenic animals (two independent lines) harboring the transgene of dominant negative RAB-8, the dendritic growth in both distal and proximal regions was dramatically reduced (S3 Fig). These results suggest that the dominant negative RAB-8 might act to block the function of both the RAB-8 and RAB-10 proteins (perhaps through inhibition of a common guanine nucleotide exchange factor) and that RAB-8 may function redundantly with RAB-10 to promote PVD morphogenesis.

To more directly test the idea that RAB-10 and RAB-8 function together in regulating PVD morphogenesis, we took advantage of a newly developed CRIPSR/Cas9-mediated conditional knock-out method and specifically disrupted the function of the *rab-10* gene in the PVD neuron and other descendants of the seam cell lineage by restricting Cas9 endonuclease expression using *nhr-81* promoter (*Pnhr-81*>*Cas9*)[41,42]. In three separate lines, approximately 5–10% of animals (11.6% (35/303), 5.1% (4/78) and 4.9% (3/61)) targeted with conditional PVD

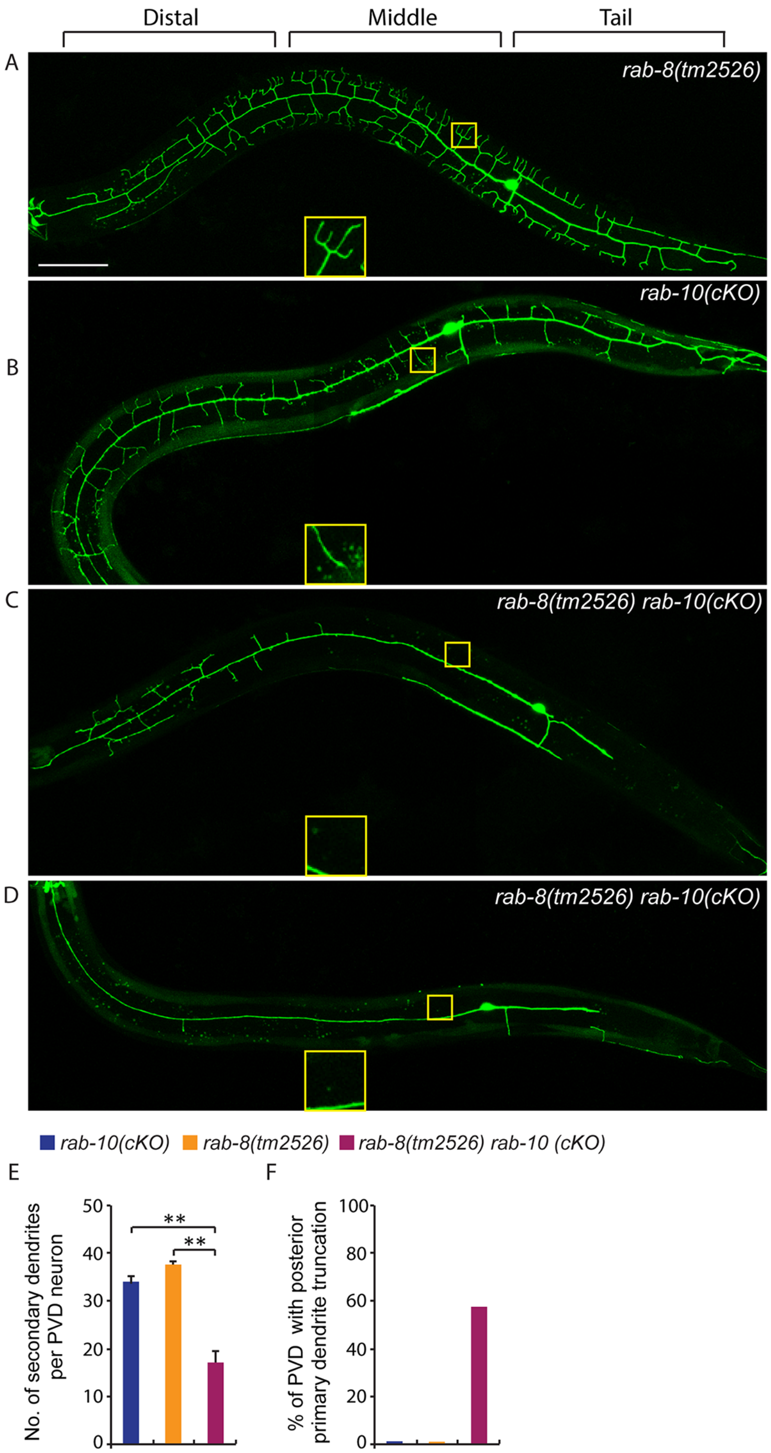

Fig 7. Deficiency in *rab-8* enhances the dendritic arborization defect of *rab-10* mutant. (A-D) Fluorescence images show the morphology of PVD neuron using the PVD>*myr-gfp* marker strain *wyIs592* in (A) *rab-8(tm2526)*, (B) *rab-10* conditional knock-out (cKO), and (C-D) *rab-10(cKO) rab-8(tm2526)*. Insets are 2.5 fold enlarged views. All images are maximum intensity projections of z-stacks. L4 or young adult stage animals were examined. Scale bar, 50 μm. (E) Quantification of number of secondary dendrites per PVD neuron in the above three genotypes (n = 35, 25 and 27, respectively). A one-way ANOVA followed by post-hoc comparisons using the Dunnett's test was used to compare single and double mutants. **: P<0.01. Error bars report ±SEM. (F) Quantification of percentage of animals showing truncated posterior primary dendrite.

doi:10.1371/journal.pgen.1005484.g007

knock-out of *rab-10* (which we refer to as *rab-10(cKO)*) generated a PVD phenotype. Animals displaying a PVD dendrite arborization defect, showed a similar phenotype as that of *rab-10* null mutants (Fig 7B). These results suggest that the CRIPSR/Cas9-mediated conditional knock-out only disrupts the *rab-10* gene in the PVD lineage in a small percentage of transgenic animals, but that when it does target *rab-10*, it completely perturbs *rab-10* function. We crossed the most penetrant *rab-10(cKO)* line into the *rab-8* deletion mutant. We hypothesized that if *rab-8* functions redundantly with *rab-10*, it should enhance the *rab-10(cKO)* PVD phenotype. From 302 animals that carried the P*nhr-81*>Cas9 and P*U6*>*rab-10*-sgRNAs transgene, 27 animals (8.9%) showed severely defective PVD dendritic arbors. We measured the total number of secondary dendrites, and found that the *rab-10(cKO) rab-8(tm2526)* animals had fewer secondary dendrites than *rab-10(cKO)* alone (Fig 7E). Further, we observed that 55.6% (15/27) of *rab-10(cKO) rab-8(tm2526)* animals had truncated posterior primary dendrites, which was rarely observed in *rab-10(cKO)* or *rab-8(tm2526)* strains (2.8% (1/35) and 0% (0/25), respectively) (Fig 7F). Taken together these results suggest that the related GTPases RAB-8 and RAB-10 function redundantly to promote dendritic morphogenesis in the PVD neuron.

Discussion

In this study, we used the two multi-dendritic PVD neurons (PVDL and PVDR) in *C. elegans*, as a model system for studying dendritic arborization. We show that the small GTPase RAB-10 is required for the growth and branching of higher-order dendrites in PVD neurons. RAB-10 localizes to Golgi and early endosomes, and its loss resulted in severe dendrite arborization defects in the proximal region of PVD neurons. Furthermore, we found that mutations in several exocyst complex components, resulted in a similar dendrite morphogenesis defect. We propose that the exocyst complex is an effector of RAB-10 and promotes docking and fusion of secretory vesicles and/or recycling endosomes, which is crucial for dendritic growth and branching (Fig 8).

rab-10 is a novel regulator of dendritic growth and branching

Previous work in *Drosophila* dendritic arborization neurons identified *rab1*, *sec23* and *sar1*, three genes that mediate ER-to-Golgi traffic, as essential regulators of membrane addition during dendritic growth [1]. This study also demonstrated that laser ablation of Golgi-outposts within the dendrites reduced dendrite arborization. Together this work strongly implicated ER-to-Golgi trafficking as well as Golgi outposts as essential for dendrite morphogenesis. These findings left open the question, however, of how membrane trafficking from the Golgi to the dendritic plasma membrane is regulated. Through a candidate-based genetic screen and analysis of the *C. elegans* multidendritic PVD neuron we have identified the small GTPase Rab10 as a post-Golgi regulator of vesicle trafficking required for dendrite morphogenesis.

Rab10 is known to regulate both Golgi-to-plasma membrane vesicle trafficking and endocytic recycling [13,22,24–26,43–45]. Our data suggest that the *C. elegans* RAB-10 protein primarily promotes post-Golgi trafficking in the PVD neuron to facilitate dendritic growth and branching. We did not observe any obvious PVD dendrite morphogenesis defects after inhibiting numerous important mediators of endocytic recycling. This includes *rme-1* and *chat-1* mutant animals and animals carrying transgenes for dominant-negative *rab-5* or *rab-11.1* GTPases [30,31]. In contrast, animals expressing a dominant-negative *rab-1*, which regulates ER-to-Golgi trafficking, showed dramatically reduced PVD dendrite branching and growth. Furthermore, animals harboring *exoc-8* and *sec-8* mutations, which encode two subunits of the exocyst complex that function to dock secretory vesicles onto fusion sites on the plasma

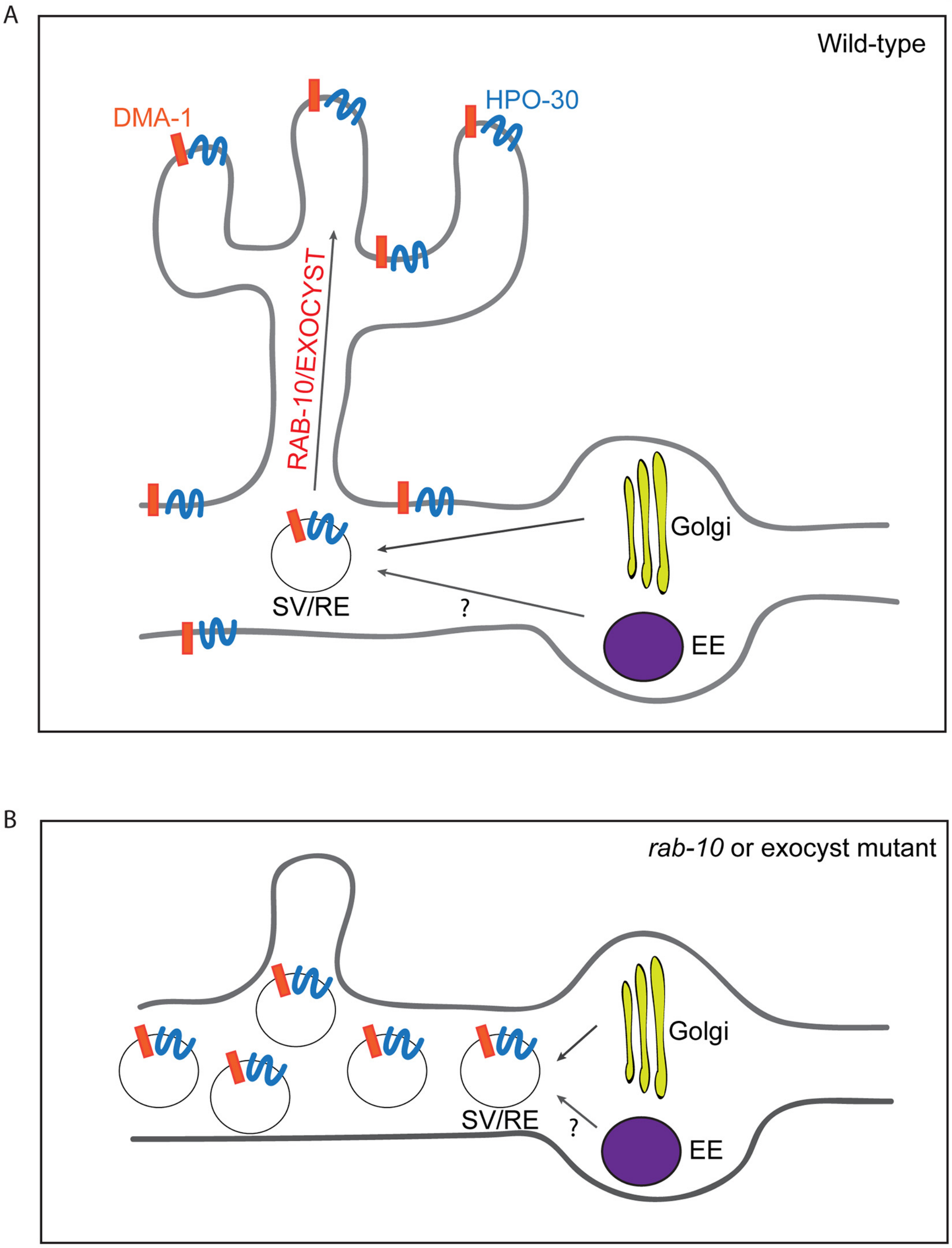

Fig 8. A summary model of how RAB-10 and exocyst complex function during dendrite arborization. (A) In wild-type animals DMA-1 and HPO-30 are transported in vesicles from the Golgi, and the trafficking of these vesicles to the proximal dendritic plasma membraneare is mediated by RAB-10 and the exocyst complex. The RAB-10 and exocyst complex might also mediate the trafficking of recycling endosome vesicles that are important for dendrite branching and stabilization. (B) In *rab-10* or exocyst mutants vesicles containing DMA-1 and HPO-30 do not traffic to the plasma membrane and accumulate in the proximal PVD dendrites. SV: secretory vesicle; RE: recycling endosome; EE: early endosome.

doi:10.1371/journal.pgen.1005484.g008

membrane, led to PVD dendrite morphogenesis defects similar to *rab-10* [17]. These data strongly implicate a function for the RAB-10 protein in mediating membrane trafficking from the secretory pathway that is crucial for dendritic branching in the PVD. Since we cannot rule out the possibility that RAB-10 might also regulate recycling of endosomes in a RME-1/RAB-5/ RAB-11.1-independent manner, we suggest it is possible that RAB-10 might also contribute to PVD morphogenesis by regulating endocytic trafficking (see Fig 8).

Higher-order dendrites are sensitive to loss of RAB-10

Although loss of *rab-10* resulted in a dramatic reduction in dendrite arborization in the PVD and FLP neurons, the absence of *rab-10* function did not affect the growth of the PVD primary dendrites or the unbranched dendrites of the OLL, AWB and AWC neuron. This might reflect a heavy reliance on post-Golgi trafficking in the PVD and FLP neurons to supply the dramatic expansion in membrane required to form highly branched dendrites. In support of this idea, a recent study in *Drosophila* revealed that Rab10 and exocyst-mediated membrane trafficking is crucial for the elaborate branching of tracheal terminal cells [44]. It is also possible that *rab-10* may transport cargoes that are necessary for the branching and growth of higher-order dendrites. Consistent with this notion, loss of *rab-10* severely affected the dendritic transport of DMA-1 and HPO-30, two dendritic specific transmembrane proteins that are required for the branching and stabilization of higher-order dendrites.

The exocyst complex promotes fusion of RAB-10 carrier vesicles and likely functions as an effector of RAB-10

The exocyst complex is a well-characterized effector of yeast Sec4p, which shares high sequence homology with RAB-10 [26]. In yeast the exocyst complex functions to target Sec4p secretory vesicles to sites of exocytosis [35]. Suggesting a similar mechanism in the PVD dendrite, we found that loss of the exocyst components *exoc-8* and *sec-8* caused a dendrite morphogenesis defect that was similar to *rab-10* mutants. In animals carrying mutations in these genes, GFP tagged DMA-1 and HPO-30 were sequestered in intracellular vesicles and the dendrite surface expression was greatly reduced. The similarity of the phenotypes suggests that the exocyst complex functions as an effector of RAB-10 to mediate trafficking of secretory vesicles to the dendritic plasma membrane. This idea is further supported by the colocalization of RAB-10 and EXOC-8 on intracellular vesicles in the PVD dendrites. Notably, compared to the PVD dendrite morphogenesis defects observed in the *rab-10* mutants, the dendrite phenotypes in *exoc-8* and *sec-8* mutants were not as severe. This could indicate that another mechanism mediates tethering, docking or fusion of RAB-10 cargo vesicles to the dendritic plasma membrane. However, it is likely that the *exoc-8* and *sec-8* mutant animals examined were not nulls for exocyst function. While the deletion allele of *exoc-8*(*ok2523*) is thought to completely remove *exoc-8* function [36], it does not lead to lethality, which is associated with loss of all other exocyst subunits. These observations suggest that loss of *exoc-8* does not completely eliminate exocyst function [46]. Further, the *sec-8*(*ok2187*) homozygotes were obtained from heterozygous mothers that likely provided maternally loaded *sec-8* mRNA or SEC-8 protein. Consistent with this idea, the removal of the exocyst component SEC-5 through ZF-1 tag-mediated degradation often resulted in a complete loss of dendritic branching. We thus suggest that the exocyst complex is the primary effector of RAB-10. Further, given that loss of SEC-5 lead to a more severe dendrite arborization defect than loss of RAB-10, it is likely the exocyst complex has functions outside of regulating RAB-10 vesicle trafficking (possibly RAB-8 vesicle trafficking, see below).

Distal dendrite arborization likely requires both *rab-8* and *rab-10*

In *rab-10* mutants, the growth and branching of more distal dendrites was minimally affected, suggesting that a *rab-10* independent mechanism exists. Notably, expressing dominant negative RAB-10 in PVD caused both distal and proximal dendrite morphogenesis defects. A possible explanation is that dominant negative RAB-10 competes for RAB activators, such as a guanine nucleotide exchange factor (GEF), that interferes with another RAB protein that is essential for distal dendrites. A strong candidate for this other RAB protein is RAB-8. Although loss of *rab-8* had no obvious effect on PVD dendrite morphogenesis, expression of a dominant negative RAB-8 in the PVD caused similar dendritic phenotype to the dominant negative RAB-10. *rab-8* is related to *rab-10*, and *rab-8* and *rab-10* function redundantly in mediating secretion in *C. elegans* germ cells [26]. Using a newly developed CRIPSR/Cas9-mediated conditional knock-out method, we found that conditional knockout of *rab-10* in a *rab-8* null mutant showed stronger dendrite arborization defect than loss of *rab-10* alone. These observations offer compelling evidence that RAB-10 and RAB-8 function redundantly to regulate PVD dendritic arborization.

RAB-10 and the exocyst complex are also required for dendrite development in flies and mammals

RAB-10 and the exocyst complex are evolutionarily conserved from *C. elegans* to humans. We observed that knock-down of *rab10* or *exo84* caused a significant reduction of the dendrite arbor in the *Drosophila* class IV dendritic arborization neuron. Knock-down of *rab10*, *exoc84* or *sec8* had no effect on the total length of the dendrites in rat cultured hippocampal neurons, but the number of dendritic spines was greatly reduced. Compared to the PVD dendrite phenotypes of *C. elegans rab-10* mutants, knock-down of *rab10* in *Drosophila* and rat neurons showed weaker dendrite morphogenesis defects. This might be due to the use of null alleles of *rab-10* in our studies of *C. elegans* compared to the RNAi strategy that led to reductions but not complete loss of *rab10* function in *Drosophila* and rat. Consistent with this, knock-out of *sec5* in *Drosophila* neurons causes more severe neurite outgrowth defect [47]. Nevertheless, our results imply that *rab-10* and exocyst-dependent membrane transport is a conserved mechanism used to build dendritic arbors and dendritic spines during neural development.

Dendrite morphogenesis defects are associated with many neurological and neurodevelopment disorders, including Autism spectrum disorders, Alzheimer's disease and Parkinson's disease [48,49]. Recovery from these diseases will rely on efficient dendrite regrowth and branching to form functional neuronal circuits. Here we identified RAB-10 and the exocyst complex as critical and conserved players during dendrite morphogenesis. Our findings reveal an evolutionarily conserved membrane transport mechanism that can efficiently supply membranes and newly synthesized transmembrane proteins to support rapid dendrite growth and morphogenesis. These findings may help in the development of new therapeutic strategies to help repair damaged neurons in human diseases and during aging.

Materials and Methods

Ethics statement

All animals were euthanized in accordance with the recommendations of the American Veterinary Medical Association and the UCSF Institutional Animal Care and Use Committee. Rats were euthanized with CO2 prior to dissection. After treatment with CO2, bilateral thoracotomy was performed to ensure the death of the animal. All experimental procedures used comply

with regulations adopted by UCSF authorities with support from the Guidelines on Euthanasia of the American Veterinary Medical Association.

C. *elegans* genetics

C. elegans was grown on OP50 *Escherichia coli*-seeded nematode growth medium agar plates at 20°C unless otherwise noted. Alleles used are as follows: Linkage group I (LGI): *rab-10* (*ok1494*), *rab-10* (*dx2*), *exoc-8* (*ok2523*), *sec-8* (*ok2187*), *exoc-7* (*ok2006*), *rab-8* (*tm2526*) and *dma-1*(*tm5159*). LGII: *sec-5* (*tm1413*), *sec-5(xn51)*, and *rrf-3*(*pk1426*). LGIV: *sec-10* (*tm3437*), and *chat-1* (*ok1681*). LGV: *ehbp-1* (*ok2140*), *rme-1* (*b1045*) and *hpo-30*(*ok2047*). Primer sequences for genotyping are available on request. RNAi experiments were performed as previously described [50], except that the *rrf-3*(*pk1246*) mutation was used to enhance the RNAi efficacy in the PVD neuron. The *dma-1* RNAi clone was obtained from Vidal RNAi feeding library and verified by DNA sequencing [51].

Transgenes and molecular biology

Standard germ line transformation by gonadal micro-injection was used to generate transgenic lines. Plasmid DNAs and fusion PCR products were used at 1–20 ng/ μl, and co-injection marker *unc-119* (+) or *Pmyo-2>mcherry* or *Pmyo-3>mcherry* or *Punc-122>rfp* or *Podr-1>gfp* was used at 1–30 ng/ μl. Chromosome integrated stable lines were generated by following gamma ray irradiation protocol [52]. Single copy transgene was generated by following a *mini-Mos*-based protocol [28]. Transgenes used are listed in S1 Table.

All the plasmids were constructed using standard molecular cloning methods. pPD49.26 and pPD95.75 were used as vectors. Coding regions of *rab-1*, *rab-8* and *rab-11.1* with dominant-negative mutations were amplified from a RAB toolkit [53]. Fusion PCR was performed as previously described [54]. Plasmids and fusion PCR product used are listed in S2 Table. Primers used are listed in S3 Table.

CRIPSR/Cas9-mediated conditional knock-out of *rab-10*

pWZ243 P*nhr-81*>Cas9 (20ng/ul), pWZ170 P*U6*>*rab-10*-sgRNA #1 (target DNA sequence was 5'GAAGAGCATGTCATACGGT3') (20ng/ul), pWZ171 P*U6*>*rab-10*-sgRNA #2 (target DNA sequence was 5'GCAATTTGAAGAGCATGTCATA3') (20ng/ul), P*myo-2>mcherry* (1ng/ul) and P*myo-3>mcherry* (5ng/ul) were injected into *wyIs592* (*ser2prom3>myr-gfp*) worms. Transgenic lines were identified and maintained based on fluorescence of the mCherry co-injection markers expressed in the pharyngeal muscles and body wall muscles. L4 and young adult stage transgenic animals were quantified for PVD dendrite arborization defects.

Quantification of dendritic arborization defect

To quantify the percentage of animals with abnormal PVD dendritic morphology (Table 1), mid-L4 to young adult stage hermaphrodite animals were anesthetized using 1mg/ml levamisole in M9 buffer, mounted on 2% agar pads and examined for PVD morphology using a compound fluorescence microscope (Carl Zeiss) with a 63X/1.4NA objective lens. Wild-type animals develop many menorah-like structures with higher-order dendrites. An animal was considered abnormal if it lacked more than four menorahs (either by lacking quaternary dendrites, tertiary dendrites or secondary dendrites) or if it had truncated primary dendrites.

To quantify PVD dendrite and axon morphology, z-stack images of PVD neurons were taken of mid- or late L4 stage animals using either a spinning disk confocal microscope (Axio Imager; Carl Zeiss) with a 40x objective lens (1.4 NA) equipped with an EM charge-coupled

device (CCD) camera (Hamamatsu Photonics) and a spinning disc confocal scan head (CSU-10; Yokogawa Electric Corporation) controlled by Micro-Manager software with 488 and 561 laser lines (for images showed in Figs 1A–1C, 2A–2B, 3A–3B, and S2A–S2D and S3A–S3B), or using a Zeiss LSM710 confocal microscope (Carl Zeiss) with a Plan-Apochromat 40X/1.3NA objective (for images showed in Figs 4B–4D, 7A–7D, S2E–S2F, S3C–S3D, S4A–S4B, S5A–S5F, S6A–S6C and S9A–S9B). The numbers of secondary, tertiary and quaternary dendrites were quantified using maximum intensity projections generated from z-stack images using ImageJ.

Time-lapse imaging of GFP::RAB-10

Mid-L4 stage hermaphrodite animals were anesthetized using 1mg/ml levamisole in M9 buffer, mounted on 2% agar pads, and then single focus plane images for GFP::RAB-10 labeled vesicles in the primary dendrites were captured using a spinning disk confocal microscope with a 100x objective lens (1.4 NA). Images were taken 1 frame per 0.8 second for 1.6 minutes (120 frames). Movies were made using ImageJ.

Colocalization analysis

Mid-L4 stage transgenic animals were anesthetized using 1mg/ml levamisole in M9 buffer, mounted on 2% agar pads and dual color images were collected using a spinning disk confocal microscope (Axio Imager; Carl Zeiss) with either a 63x (for images showed in Figs 2D–2E and 6D–6E) or 100x (for images showed in Fig 6A) objective lens (1.4 NA). Colocalization between RAB-10 and RAB-5, FAPP1-PH and EXOC-8 was quantified using Coloc 2, a Fiji's plugin for colocalization analysis (http://fiji.sc/Coloc_2). The Pearson correlation coefficient index is shown for each group.

Quantification of intensity of DMA-1::GFP and HPO-30::GFP

Z-stacks of *ser2prom3*>DMA-1::GFP and *ser2prom3*>HPO-30::GFP fluorescence in distal, middle and tail region of mid-L4 stage worms were taken using a spinning disk confocal microscope (Axio Imager; Carl Zeiss) with a 100x Plan-Aprochromat objective lens (1.4 NA). Fluorescence across the primary dendrites was quantified using the "measure" function of ImageJ software from single focal plane images. Background subtraction levels were determined from regions outside of the worms, lacking any GFP signal.

Quantification of DMA-1::GFP, HPO-30::GFP and GFP::RAB-10 vesicular units

Z-stacks of *ser2prom3*>DMA-1::GFP and *ser2prom3*>HPO-30::GFP fluorescence in distal, middle and tail region of mid-L4 stage worms were taken using a spinning disk confocal microscope (Axio Imager; Carl Zeiss) with a 100x Plan-Aprochromat objective lens (1.4 NA). Number of vesicular units (either single vesicles or vesicle clusters) was quantified from images of maximum intensity projections generated from z-stack images using ImageJ. The size of each image was 76.8μm x 76.8 μm.

Z-stacks of *ser2prom3*>GFP::RAB-10 fluorescence in middle region of mid-L4 stage worms were taken using a spinning disk confocal microscope (Axio Imager; Carl Zeiss) with a 63x Plan-Aprochromat objective lens (1.4 NA). Number of vesicular units (either single vesicles or vesicle clusters) was quantified from maximum intensity projections generated from z-stacks using ImageJ. The size of each image was 88.1μm x 88.1 μm.

Drosophila strains and quantification of the dendritic arbor of class IV neurons

The UAS-Gal4 system was used to express RNAi in the class IV neurons of Drosophila larvae. UAS-RNAi lines are available from Bloomington Stock Center: y[1] v[1]; P{y[+t7.7] v[+t1.8] = TRiP.JF02058}attP2 (BL 26289), y[1] v[1]; P{y[+t7.7] v[+t1.8] = TRiP.JF03139}attP2 (BL 28712) and from the Vienna Drosophila Resource Center: w[1118]; P{GD16778}v46791/CyO; (v46791), w[1118]; P{GD11816}v30112/TM3; (v30112). UAS- RNAi lines were crossed to ppktdGFP; ppkgal4, UAS-dcr2 virgins. Larvae resulting from this cross were imaged at third larval instar. The dendritic arbor was quantified as described [55].

shRNA-mediated knockdown in rat cultured hippocampal neurons

Hippocampal neurons cultured from E19 Long-Evans rats (Charles River Lab) were plated at a density of 200,000 neurons per 18 mm acid treated glass coverslips (Fisher) coated with 0.06 mg/ml poly-D-lysine (Sigma) and 2.5 ug/ml Laminin (Sigma). Neurons were plated using plating media (Modified Eagle Medium + 10% Fetal Bovine Serum (Hyclone), 0.45% dextrose, 0.11 mg/ml sodium pyruvate, 2mM glutamine, Reagents from UCSF cell culture facility). Cultures were transferred to maintenance media (Neurobasal Media, Invitrogen+ 0.5mM Glutamine +1X B27, Invitrogen and Penicillin/Streptomycin) 4 hours post plating. Half of the media was replaced with fresh media every 4 days.

All shRNA constructs were made in the pLentilox3.7 vector backbone using *TTCAAGAGA* as the loop sequence. Sequences used for constructing the shRNAs are: 1) Rab10 (NM_017359.2 Start position: 2373) "TTGACTCTATCATTGTTTA" 2) Exoc84 (NM_139043.1 Start position: 310) "CGCAGAACCTGAAGCGCAA" 3) Sec8 (NM_053875.1 Start position: 887) "CCGTTAAAGCCATTAAAGA". ShRNA transfections were performed using Lipofectamine-2000 (Invitrogen) following manufacturer's guidelines. Transfections were done at DIV7 and DIV14 and fixed 48 hours post transfection at DIV9 and DIV16, respectively using 4% PFA+4% sucrose at room temperature for 15 min. We did not observe toxicity or neuronal death in any of the shRNA treatment conditions. Neurons were blocked in blocking buffer (10% normal donkey serum+ 0.2M glycine+ 0.1% triton-x100 in Phosphate buffer saline) for an hour followed by overnight incubation with primary antibodies (mouse anti GFP, from Roche and rabbit anti-MAP2 from Chemicon). Coverslips were washed thrice in PBS and incubated for 2 hours with secondary antibodies (mouse Alexa 488 and Rabbit Alexa 568, from Jackson ImmunoResearch) followed by three washes in PBS before mounting onto slides for analysis using Fluromont mounting media from EMS).

Neuronal morphology quantification in rat cultured neurons

Imaging of rat hippocampal neurons was performed on Leica Sp5 scanning confocal microscope with Laser lines 488 and 561 using a 40X NA 1.25 objective with zoom 0 for dendrite length and zoom 3.0 for dendritic spine imaging. Confocal image stacks were acquired at 1024X1024 pixels with 0.4micron z spacing between two frames such that the entire depth of the neuron/dendrite was imaged. Analysis was performed on maximum projected image stacks using ImageJ. ShRNA transfected cells were identified by GFP expression. Neurite length was calculated using the 'Measure' function of ImageJ and dendrite density was calculated by manually counting the number of spines per 100 microns of dendrite length.

Statistical analysis

One-way ANOVA followed by post-hoc comparisons using the Dunnett's test was used for all the figures, except S11 Fig (one-way ANOVA followed by post-hoc comparisons using the Holm-Sidak test was used in this figure). Error bar means standard error of mean (SEM) for all the figures except S11 Fig (error bar means standard deviation in this figure).

Supporting Information

S1 Fig. Axonal growth is not affected in *rab-10*, *exoc-8* and *sec-8* mutants. Length of axons was quantified for wild-type, *rab-10(ok1494)*, *rab-10(dx2)*, *exoc-8(ok2523)* and *sec-8(ok2187)* worms from maximum intensity projections of z-stacks using Image J. Axons were visualized using the *wdIs51* strain. At least 15 animals were quantified for each genotype. A one-way ANOVA was used to compare wild-type and mutant animals. Error bars report ±SEM. ns: not significant.
(TIF)

S2 Fig. Loss of *rab-10* affects the dendritic growth of the multidendritic FLP but not the unbranched dendrites of the OLL, AWB and AWC neurons. Morphology of (A, B) FLP neuron, (C, D) OLL neuron and (E, F) AWB and AWC neurons in wild-type (A, C, and E) and *rab-10(ok1494)* mutant animals (B, D and F) are shown. All images are maximum z projections. L4 or young adult stage animals were examined. Scale bar, 20 μm.
(TIF)

S3 Fig. Dominant negative RAB-10 and RAB-8 reduce distal and proximal dendrite branching. (A-D) Images showing the morphology of PVD neuron in (A, B) transgenic animals carrying *Is*[*ser2prom3*>*gfp*::*rab-10 DN*] and (C, D) transgenic animals carrying *Ex*[*ser2-prom3*>*rab-8 DN*]. PVD morphology was visualized using the PVD>*gfp* marker strain *wdIs51*. All images are maximum z projections. L4 stage animals were examined. Scale bar, 50 μm. (E) Quantification of animals with wild-type or defective PVD dendrite morphology. Two independent lines were quantified for dominant negative RAB-10 and RAB-8, respectively. At least 30 worms were examined. D+P defect means that dendrite arborization in both distal and proximal regions were reduced (shown in A and C). Proximal defect means that only dendrites in the proximal region were affected (shown in B and D). No defect means the morphology of PVD dendrite arbors were indistinguishable from that of wild-type worms.
(TIF)

S4 Fig. Over-expressing constitutively active RAB-10 does not induce dendrite overgrowth. (A-B) Fluorescence images show the morphology of PVD neuron using the PVD>*myr-gfp* marker strain *wyIs592* in (A) wild-type and (B) GFP::RAB-10 Q68L overexpression. Both images are maximum intensity projections of z-stacks. L4 stage animals were examined. Scale bar, 50 μm. (C-E) Quantification of number of (C) secondary dendrites, (D) tertiary dendrites and (E) quaternary dendrites per PVD neuron in the above three genotypes (n = 12 for each strain). A one-way ANOVA was used to compare wild-type and RAB-10 Q68L over-expressing lines. Error bars report ±SEM. ns: not significant.
(TIF)

S5 Fig. GFP::RAB-10, DMA-1::GFP and HPO-30::GFP are functional. (A-F) Fluorescence images show the morphology of PVD neuron using the PVD>GFP marker strain *wdIs51* in (A) *rab-10*(*ok1494*), (B) *rab-10*(*ok1494*); *Ti*[*ser2prom3*>*gfp*::*rab-10*], (C) *dma-1*(*tm5159*), (D) *dma-1*(*tm5159*); *Is*[*ser2prom3*>*dma-1*::*gfp*], (E) *hpo-30*(*ok2047*), and (F) *hpo-30*(*ok2047*); *Is* [*ser2prom3*>*hpo-30*::*gfp*]. All images are maximum intensity projections of z-stacks. L4 or

young adult stage animals were examined. Scale bars, 50 μm. (G) Quantification of cell-autonomous rescue of dendritic morphogenesis defect by cell-specific expression of *gfp*::*rab-10*, *dma-1*::*gfp* and *hpo-30*::*gfp* transgenes. At least 30 animals were quantified for each genotype.
(TIF)

S6 Fig. Dominant negative RAB-1 in PVD reduces dendrite arborization. (A-C) PVD morphology was visualized using the strain *wdIs51*. Fluorescence images show the morphology of PVD neuron in (A) wild type, (B and C) *ser2prom3>rab-1 DN* transgenic animals. See Table 1 for quantification of defect. All images are maximum intensity projections of z-stacks. L4 or young adult stage animals were examined. Scale bar, 50 μm.
(TIF)

S7 Fig. Loss of *rab-10* and *exoc-8* affect HPO-30 transport to the dendritic membranes. (A-I) Fluorescence images show subcellular localization of HPO-30::GFP in (A-C) wild-type, (D-F) *rab-10* (*ok1494*) and (G-I) *exoc-8* (*ok2523*) in the distal, middle and tail regions, respectively. All images are maximum intensity projections of z-stacks. L4 stage animals were examined. Insets are enlarged 2.5 fold. Scale bar, 10 μm. (J) Quantification of number of HPO-30::GFP vesicles/vesicle clusters per area (76.8μm x 76.8μm). 10 animals at the L4 stage were quantified for each genotype. (K) Quantification of the intensity of HPO-30::GFP at the surface of the primary dendrites in wild-type, *rab-10* (*ok1494*) and *exoc-8* (*ok2523*) mutant worms in the distal, middle, and tail regions, respectively. 10 animals at the L4 stage were quantified for each genotype. A one-way ANOVA followed by post-hoc comparisons using the Dunnett's test was used to compare wild-type and mutant animals. **: $P<0.01$. Error bars report ±SEM.
(TIF)

S8 Fig. DMA-1::GFP labeled vesicles accumulate in higher-order dendrites of *rab-10* and *exoc-8* mutants. Number of vesicles or vesicle clusters labeled by DMA-1::GFP was quantified. At least 60 secondary dendrites were quantified for each genotype. A one-way ANOVA followed by post-hoc comparisons using the Dunnett's test was used to compare wild-type and mutant animals. **: $P<0.01$. Error bars report ±SEM.
(TIF)

S9 Fig. Knock-down of *dma-1* disrupts distal dendrite arborization in *rab-10* mutants. (A-B) Fluorescence images show the morphology of PVD neuron using the PVD>*gfp* marker strain *wdIs51* in *rab-10(ok1494)*; *rrf-3(pk1426)* fed with (A) control RNAi strain (harboring L4440 empty vector) and (B) *dma-1* RNAi strain. Both images are maximum intensity projections of z-stacks. L4 or young adult stage animals were examined. Scale bar, 50 μm.
(TIF)

S10 Fig. Vesicular localization of EXOC-8::GFP is not altered in *rab-10* loss-of-function mutants. (A-B) Fluorescence images showing EXOC-8::GFP in (A) wild-type and (B) *rab-10 (ok1494)* mutant. Both images are maximum intensity projections of z-stacks. L4 or young adult stage animals were examined. Scale bars, 10 μm. The inset images are enlarged views (2.5 fold) of the regions indicated by the boxes. Arrows indicate vesicles labeled by EXOC-8::GFP reporter.
(TIF)

S11 Fig. *Drosophila* RAB10 and EXO84 mediate dendritic morphogenesis. (A-F) Fluorescence images showing expression of a reporter *ppk-tdgfp* for the *Drosophila* class IV dendritic arborization neurons in (A and B) wild type, (C and D) *rab10* RNAi and (E and F) *exo84* RNAi treated animals. The images shown in (B, D and F) are magnified 2.5 fold from the boxed regions shown directly above. Scale bars, 100 μm. (G-H) Quantification of the number of (G) end points and (H) total dendritic arbor in wild type, *rab10* RNAi (2 independent lines) and

exo84 RNAi (2 independent lines) in *Drosophila* class IV dendritic arborization neurons. At least 7 neurons were quantified for each genotype. A one-way ANOVA followed by post-hoc comparisons using the Holm-Sidak test was used to compare wild-type and mutant animals. *: P<0.05; ns: not significant. Error bars report ±SD.
(TIF)

S12 Fig. Mammalian RAB10, SEC8 and EXO84 promote formation of the dendritic spines in rat hippocampal neurons. (A-H) Confocal fluorescence maximum projected images of cultured rat hippocampal neurons transfected with (A and B) control shRNA, (C and D) shRNA against *Rab10* (E and F) shRNA against *Sec8* and (G and H) shRNA against *Exo84*. Immunolocalization was conducted using antibodies against GFP (neuron, green) and MAP2 (dendrite, red). Scale bars, 20 μm. (I-J) Quantification of (I) total dendrite length at DIV9 and (J) dendritic spine density at DIV16 in control shRNA, *Rab10* shRNA, *Sec8* shRNAi and *Exo84* shRNA transfected rat hippocampal neurons. 10 neurons were quantified for each genotype. A one-way ANOVA followed by post-hoc comparisons using the Dunnett's test was used to compare wild-type and mutant animals. ns: not significant; **: P<0.01. Error bars report ±SEM.
(TIF)

S1 Movie. GFP::RAB-10 labeled vesicles move bi-directionally along the dendrites. The time-lapse shows GFP::RAB-10 labeled vesicles moving bi-directionally in the PVD dendrites. Frames were collected every 0.8 sec. for a total of 96 sec using a spinning disk confocal (CSU-10; Yokogawa Electri Corporation). The movie was generated using 7 frames per second. Scale bar: 10μm.
(AVI)

S1 Table. Transgenes used in this study.
(DOCX)

S2 Table. Plasmids and fusion PCR products used in this study.
(DOCX)

S3 Table. Primers used in this study.
(DOCX)

Acknowledgments

Some strains were provided by the CGC, which is funded by NIH Office of Research Infrastructure Programs (P40 OD010440), and the MITANI Lab through the National Bio-Resource Project of the MEXT, Japan. We thank Dr. Kang Shen (Stanford University) for sharing results prior publication and reagents, Drs. Jeremy Nance (New York University), Johnny Fares (University of Arizona), Maria Gallegos (California State University), Barth Grant (Rutgers University), Jordan Ward (University of California, San Francisco), Christian Frøkjær-Jensen (Stanford Univeristy) and Xiaochen Wang (National Institute of Biological Sciences, Beijing) for reagents, and Qiuyi Chi (Duke University) for technical assistance. We also thank Drs. Nina T. Sherwood, David Miller III, Zheng Wang, Baohui Chen and members of our laboratories for discussions.

Author Contributions

Conceived and designed the experiments: WZ SY LD YNJ DRS. Performed the experiments: WZ SY LD. Analyzed the data: WZ SY LD. Contributed reagents/materials/analysis tools: WZ SY LD. Wrote the paper: WZ SY LD YNJ DRS.

References

1. Ye B, Zhang Y, Song W, Younger SH, Jan LY, et al. (2007) Growing dendrites and axons differ in their reliance on the secretory pathway. Cell 130: 717–729. PMID: 17719548
2. Satoh D, Sato D, Tsuyama T, Saito M, Ohkura H, et al. (2008) Spatial control of branching within dendritic arbors by dynein-dependent transport of Rab5-endosomes. Nat Cell Biol 10: 1164–1171. doi: 10.1038/ncb1776 PMID: 18758452
3. Lee MC, Miller EA, Goldberg J, Orci L, Schekman R (2004) Bi-directional protein transport between the ER and Golgi. Annu Rev Cell Dev Biol 20: 87–123. PMID: 15473836
4. Lazo OM, Gonzalez A, Ascano M, Kuruvilla R, Couve A, et al. (2013) BDNF regulates Rab11-mediated recycling endosome dynamics to induce dendritic branching. J Neurosci 33: 6112–6122. doi: 10.1523/JNEUROSCI.4630-12.2013 PMID: 23554492
5. Way JC, Chalfie M (1989) The mec-3 gene of Caenorhabditis elegans requires its own product for maintained expression and is expressed in three neuronal cell types. Genes Dev 3: 1823–1833. PMID: 2576011
6. Chatzigeorgiou M, Yoo S, Watson JD, Lee WH, Spencer WC, et al. (2010) Specific roles for DEG/ENaC and TRP channels in touch and thermosensation in C. elegans nociceptors. Nat Neurosci 13: 861–868. doi: 10.1038/nn.2581 PMID: 20512132
7. Li W, Kang L, Piggott BJ, Feng Z, Xu XZ (2011) The neural circuits and sensory channels mediating harsh touch sensation in Caenorhabditis elegans. Nat Commun 2: 315. doi: 10.1038/ncomms1308 PMID: 21587232
8. Smith CJ, Watson JD, Spencer WC, O'Brien T, Cha B, et al. (2010) Time-lapse imaging and cell-specific expression profiling reveal dynamic branching and molecular determinants of a multi-dendritic nociceptor in C. elegans. Dev Biol 345: 18–33. doi: 10.1016/j.ydbio.2010.05.502 PMID: 20537990
9. Liu OW, Shen K (2012) The transmembrane LRR protein DMA-1 promotes dendrite branching and growth in C. elegans. Nat Neurosci 15: 57–63.
10. Dong X, Liu OW, Howell AS, Shen K (2013) An extracellular adhesion molecule complex patterns dendritic branching and morphogenesis. Cell 155: 296–307. doi: 10.1016/j.cell.2013.08.059 PMID: 24120131
11. Salzberg Y, Diaz-Balzac CA, Ramirez-Suarez NJ, Attreed M, Tecle E, et al. (2013) Skin-derived cues control arborization of sensory dendrites in Caenorhabditis elegans. Cell 155: 308–320. doi: 10.1016/j.cell.2013.08.058 PMID: 24120132
12. Smith CJ, O'Brien T, Chatzigeorgiou M, Spencer WC, Feingold-Link E, et al. (2013) Sensory neuron fates are distinguished by a transcriptional switch that regulates dendrite branch stabilization. Neuron 79: 266–280. doi: 10.1016/j.neuron.2013.05.009 PMID: 23889932
13. Wang T, Liu Y, Xu XH, Deng CY, Wu KY, et al. (2011) Lgl1 activation of rab10 promotes axonal membrane trafficking underlying neuronal polarization. Dev Cell 21: 431–444. doi: 10.1016/j.devcel.2011.07.007 PMID: 21856246
14. Liu Y, Xu XH, Chen Q, Wang T, Deng CY, et al. (2013) Myosin Vb controls biogenesis of post-Golgi Rab10 carriers during axon development. Nat Commun 4: 2005. doi: 10.1038/ncomms3005 PMID: 23770993
15. Deng CY, Lei WL, Xu XH, Ju XC, Liu Y, et al. (2014) JIP1 mediates anterograde transport of Rab10 cargos during neuronal polarization. J Neurosci 34: 1710–1723. doi: 10.1523/JNEUROSCI.4496-13.2014 PMID: 24478353
16. Xu XH, Deng CY, Liu Y, He M, Peng J, et al. (2014) MARCKS regulates membrane targeting of Rab10 vesicles to promote axon development. Cell Res 24: 576–594. doi: 10.1038/cr.2014.33 PMID: 24662485
17. He B, Guo W (2009) The exocyst complex in polarized exocytosis. Curr Opin Cell Biol 21: 537–542. doi: 10.1016/j.ceb.2009.04.007 PMID: 19473826
18. Frische EW, Pellis-van Berkel W, van Haaften G, Cuppen E, Plasterk RH, et al. (2007) RAP-1 and the RAL-1/exocyst pathway coordinate hypodermal cell organization in Caenorhabditis elegans. EMBO J 26: 5083–5092. PMID: 17989692
19. Babbey CM, Bacallao RL, Dunn KW (2010) Rab10 associates with primary cilia and the exocyst complex in renal epithelial cells. Am J Physiol Renal Physiol 299: F495–506. doi: 10.1152/ajprenal.00198.2010 PMID: 20576682
20. Sano H, Eguez L, Teruel MN, Fukuda M, Chuang TD, et al. (2007) Rab10, a target of the AS160 Rab GAP, is required for insulin-stimulated translocation of GLUT4 to the adipocyte plasma membrane. Cell Metab 5: 293–303. PMID: 17403373

21. Inoue M, Chang L, Hwang J, Chiang SH, Saltiel AR (2003) The exocyst complex is required for targeting of Glut4 to the plasma membrane by insulin. Nature 422: 629–633. PMID: 12687004

22. Babbey CM, Ahktar N, Wang E, Chen CC, Grant BD, et al. (2006) Rab10 regulates membrane transport through early endosomes of polarized Madin-Darby canine kidney cells. Mol Biol Cell 17: 3156–3175. PMID: 16641372

23. Schuck S, Gerl MJ, Ang A, Manninen A, Keller P, et al. (2007) Rab10 is involved in basolateral transport in polarized Madin-Darby canine kidney cells. Traffic 8: 47–60. PMID: 17132146

24. Chen CC, Schweinsberg PJ, Vashist S, Mareiniss DP, Lambie EJ, et al. (2006) RAB-10 is required for endocytic recycling in the Caenorhabditis elegans intestine. Mol Biol Cell 17: 1286–1297. PMID: 16394106

25. Glodowski DR, Chen CC, Schaefer H, Grant BD, Rongo C (2007) RAB-10 regulates glutamate receptor recycling in a cholesterol-dependent endocytosis pathway. Mol Biol Cell 18: 4387–4396. PMID: 17761527

26. Shi A, Chen CC, Banerjee R, Glodowski D, Audhya A, et al. (2010) EHBP-1 functions with RAB-10 during endocytic recycling in Caenorhabditis elegans. Mol Biol Cell 21: 2930–2943. doi: 10.1091/mbc.E10-02-0149 PMID: 20573983

27. Arakawa H, Kudo H, Batrak V, Caldwell RB, Rieger MA, et al. (2008) Protein evolution by hypermutation and selection in the B cell line DT40. Nucleic Acids Res 36: e1. PMID: 18073192

28. Frokjaer-Jensen C, Davis MW, Sarov M, Taylor J, Flibotte S, et al. (2014) Random and targeted transgene insertion in Caenorhabditis elegans using a modified Mos1 transposon. Nat Methods 11: 529–534. doi: 10.1038/nmeth.2889 PMID: 24820376

29. Guo P, Hu T, Zhang J, Jiang S, Wang X (2010) Sequential action of Caenorhabditis elegans Rab GTPases regulates phagolysosome formation during apoptotic cell degradation. Proc Natl Acad Sci U S A 107: 18016–18021. doi: 10.1073/pnas.1008946107 PMID: 20921409

30. Grant B, Zhang Y, Paupard MC, Lin SX, Hall DH, et al. (2001) Evidence that RME-1, a conserved C. elegans EH-domain protein, functions in endocytic recycling. Nat Cell Biol 3: 573–579. PMID: 11389442

31. Chen B, Jiang Y, Zeng S, Yan J, Li X, et al. (2010) Endocytic sorting and recycling require membrane phosphatidylserine asymmetry maintained by TAT-1/CHAT-1. PLoS Genet 6: e1001235. doi: 10.1371/journal.pgen.1001235 PMID: 21170358

32. Woodman PG (2000) Biogenesis of the sorting endosome: the role of Rab5. Traffic 1: 695–701. PMID: 11208157

33. Ullrich O, Reinsch S, Urbe S, Zerial M, Parton RG (1996) Rab11 regulates recycling through the pericentriolar recycling endosome. J Cell Biol 135: 913–924. PMID: 8922376

34. Grosshans BL, Ortiz D, Novick P (2006) Rabs and their effectors: achieving specificity in membrane traffic. Proc Natl Acad Sci U S A 103: 11821–11827. PMID: 16882731

35. Guo W, Roth D, Walch-Solimena C, Novick P (1999) The exocyst is an effector for Sec4p, targeting secretory vesicles to sites of exocytosis. EMBO J 18: 1071–1080. PMID: 10022848

36. Jiu Y, Jin C, Liu Y, Holmberg CI, Jantti J (2012) Exocyst subunits Exo70 and Exo84 cooperate with small GTPases to regulate behavior and endocytic trafficking in C. elegans. PLoS One 7: e32077. doi: 10.1371/journal.pone.0032077 PMID: 22389680

37. Armenti ST, Lohmer LL, Sherwood DR, Nance J (2014) Repurposing an endogenous degradation system for rapid and targeted depletion of C. elegans proteins. Development 141: 4640–4647. doi: 10.1242/dev.115048 PMID: 25377555

38. Grindstaff KK, Yeaman C, Anandasabapathy N, Hsu SC, Rodriguez-Boulan E, et al. (1998) Sec6/8 complex is recruited to cell-cell contacts and specifies transport vesicle delivery to the basal-lateral membrane in epithelial cells. Cell 93: 731–740. PMID: 9630218

39. Tsuboi T, Ravier MA, Xie H, Ewart MA, Gould GW, et al. (2005) Mammalian exocyst complex is required for the docking step of insulin vesicle exocytosis. J Biol Chem 280: 25565–25570. PMID: 15878854

40. Pereira-Leal JB, Seabra MC (2000) The mammalian Rab family of small GTPases: definition of family and subfamily sequence motifs suggests a mechanism for functional specificity in the Ras superfamily. J Mol Biol 301: 1077–1087. PMID: 10966806

41. Shen Z, Zhang X, Chai Y, Zhu Z, Yi P, et al. (2014) Conditional knockouts generated by engineered CRISPR-Cas9 endonuclease reveal the roles of coronin in C. elegans neural development. Dev Cell 30: 625–636. doi: 10.1016/j.devcel.2014.07.017 PMID: 25155554

42. Miyabayashi T, Palfreyman MT, Sluder AE, Slack F, Sengupta P (1999) Expression and function of members of a divergent nuclear receptor family in Caenorhabditis elegans. Dev Biol 215: 314–331. PMID: 10545240

43. Lerner DW, McCoy D, Isabella AJ, Mahowald AP, Gerlach GF, et al. (2013) A Rab10-dependent mechanism for polarized basement membrane secretion during organ morphogenesis. Dev Cell 24: 159–168. doi: 10.1016/j.devcel.2012.12.005 PMID: 23369713

44. Jones TA, Nikolova LS, Schjelderup A, Metzstein MM (2014) Exocyst-mediated membrane trafficking is required for branch outgrowth in Drosophila tracheal terminal cells. Dev Biol 390: 41–50. doi: 10.1016/j.ydbio.2014.02.021 PMID: 24607370

45. Chen S, Li L, Li J, Liu B, Zhu X, et al. (2014) SEC-10 and RAB-10 coordinate basolateral recycling of clathrin-independent cargo through endosomal tubules in Caenorhabditis elegans. Proc Natl Acad Sci U S A 111: 15432–15437. doi: 10.1073/pnas.1408327111 PMID: 25301900

46. Jiu Y, Hasygar K, Tang L, Liu Y, Holmberg CI, et al. (2014) par-1, atypical pkc, and PP2A/B55 sur-6 are implicated in the regulation of exocyst-mediated membrane trafficking in Caenorhabditis elegans. G3 (Bethesda) 4: 173–183.

47. Murthy M, Teodoro RO, Miller TP, Schwarz TL (2010) Sec5, a member of the exocyst complex, mediates Drosophila embryo cellularization. Development 137: 2773–2783. doi: 10.1242/dev.048330 PMID: 20630948

48. Einstein G, Buranosky R, Crain BJ (1994) Dendritic pathology of granule cells in Alzheimer's disease is unrelated to neuritic plaques. J Neurosci 14: 5077–5088. PMID: 8046469

49. McNeill TH, Brown SA, Rafols JA, Shoulson I (1988) Atrophy of medium spiny I striatal dendrites in advanced Parkinson's disease. Brain Res 455: 148–152. PMID: 3416180

50. Aguirre-Chen C, Bulow HE, Kaprielian Z (2011) C. elegans bicd-1, homolog of the Drosophila dynein accessory factor Bicaudal D, regulates the branching of PVD sensory neuron dendrites. Development 138: 507–518. doi: 10.1242/dev.060939 PMID: 21205795

51. Rual JF, Ceron J, Koreth J, Hao T, Nicot AS, et al. (2004) Toward improving Caenorhabditis elegans phenome mapping with an ORFeome-based RNAi library. Genome Res 14: 2162–2168. PMID: 15489339

52. Inoue T, Sherwood DR, Aspock G, Butler JA, Gupta BP, et al. (2002) Gene expression markers for Caenorhabditis elegans vulval cells. Mech Dev 119 Suppl 1: S203–209. PMID: 14516686

53. Gallegos ME, Balakrishnan S, Chandramouli P, Arora S, Azameera A, et al. (2012) The C. elegans rab family: identification, classification and toolkit construction. PLoS One 7: e49387. doi: 10.1371/journal.pone.0049387 PMID: 23185324

54. Hobert O (2002) PCR fusion-based approach to create reporter gene constructs for expression analysis in transgenic C. elegans. Biotechniques 32: 728–730. PMID: 11962590

55. Iyer EP, Iyer SC, Sullivan L, Wang D, Meduri R, et al. (2013) Functional genomic analyses of two morphologically distinct classes of Drosophila sensory neurons: post-mitotic roles of transcription factors in dendritic patterning. PLoS One 8: e72434. doi: 10.1371/journal.pone.0072434 PMID: 23977298
